# Turnover of southern cypresses in the post‐Gondwanan world: extinction, transoceanic dispersal, adaptation and rediversification

**DOI:** 10.1111/nph.15561

**Published:** 2018-11-23

**Authors:** Michael D. Crisp, Lyn G. Cook, David M. J. S. Bowman, Meredith Cosgrove, Yuji Isagi, Shota Sakaguchi

**Affiliations:** ^1^ Research School of Biology The Australian National University RN Robertson Building, 46 Sullivans Creek Road Acton (Canberra) ACT 2601 Australia; ^2^ School of Biological Sciences The University of Queensland Brisbane Qld 4072 Australia; ^3^ School of Natural Sciences The University of Tasmania Private Bag 55 Hobart Tas 7001 Australia; ^4^ Graduate School of Agriculture Kyoto University Kyoto 606‐8502 Japan; ^5^ Graduate School of Human and Environmental Studies Kyoto University Kyoto 606‐8501 Japan

**Keywords:** biome shift, *Callitris*, conifers, extinction, fossils, long‐distance dispersal, serotiny, vicariance

## Abstract

- Cupressaceae subfamily Callitroideae has been an important exemplar for vicariance biogeography, but its history is more than just disjunctions resulting from continental drift. We combine fossil and molecular data to better assess its extinction and, sometimes, rediversification after past global change.
- Key fossils were reassessed and their phylogenetic placement for calibration was determined using trait mapping and Bayes Factors. Five vicariance hypotheses were tested by comparing molecular divergence times with the timing of tectonic rifting. The role of adaptation to fire (serotiny) in its spread across a drying Australia was tested for *Callitris*.
- Our findings suggest that three transoceanic disjunctions within the Callitroideae probably arose from long‐distance dispersal. A signature of extinction, centred on the end‐Eocene global climatic chilling and drying, is evident in lineages‐through‐time plots and in the fossil record. *Callitris*, the most diverse extant callitroid genus, suffered extinctions but surviving lineages adapted and re‐radiated into dry, fire‐prone biomes that expanded in the Neogene. Serotiny, a key adaptation to fire, likely evolved in *Callitris* coincident with the biome shift.
- Both extinction and adaptive shifts have probably played major roles in this chronicle of turnover and renewal, but better understanding of biogeographical history requires improved taxonomy of fossils.

## Introduction

The cypress family of conifers (Cupressaceae) has a rich and ancient fossil record going back > 200 Myr to Pangaea. Today it is widespread across both the northern and southern continents and therefore is an excellent model for testing biogeographical hypotheses about the relative roles of continental drift and long‐distance dispersal and establishment (LDDE) in explaining transoceanic disjunctions (Crisp *et al*., 2009). The cypress fossil record is exceptional (Hill & Brodribb, 1999; Stockey *et al*., 2005), better than for most plant families, providing tangible evidence for the presence of lineages in different places at different times. This record also provides multiple calibration points for molecular clock analyses.

A recent biogeographical modelling study (Mao *et al*., 2012) explained the distribution of cypresses by overland migration across the supercontinents (Pangaea, Laurasia and Gondwana) before they rifted apart and broke up. That is, they concluded that phylogeny and distribution of the family reflect patterns consistent with vicariance rather than LDDE. However, there were assumptions and omissions in their analysis that might have affected the accuracy of their analysis of the Southern Hemisphere subfamily Callitroideae (callitroids). For example, they treated Australia and Zealandia as a single landmass until the present, whereas they rifted apart *c*. 80–65 million yr ago (Ma) (McLoughlin, 2001; Mortimer *et al*., 2017), well before two inferred transoceanic disjunctions in the callitroids. Also, they omitted some key callitroid fossils that are relevant to molecular clock calibration and biogeography, and they sampled fewer than half of the living species of *Callitris* s.l., which is the largest callitroid group. *Callitris* is defined broadly herein to include *Actinostrobus* and *Neocallitropsis*, which have been formally synonymized (Piggin & Bruhl, 2010; Byng, 2015).

During the Oligocene, following initiation of global climate change at the end of the Eocene, there was extensive extinction of conifers including callitroids, as indicated by both fossil (Niklas, 1997) and phylogenetic evidence (Crisp & Cook, 2011). This extinction particularly impacted Australia, where most of the fire‐sensitive, ever‐wet adapted Southern Hemisphere cypresses had disappeared from the fossil record by the early Miocene (Hill & Brodribb, 1999; Hill, 2004; Hill & Brodribb, 2006; see also Fig. 2) as the continent dried and became dominated by fire. Exceptionally, *Callitris* survived and rediversified into multiple habitats throughout Australia, including the arid zone, and into New Caledonia (NC). *Callitris* is sensitive to crown fire but its seed cones have the morphological characteristics of serotiny, a syndrome which is hypothesized to be adaptive to crown‐fire regimes (Lamont *et al*., 1991). However, it is unclear whether serotiny in *Callitris* evolved in response to some other selective pressure (exaptation: Bradshaw *et al*., 2011) or was a direct response to pyric selection after transition to a fire‐dominated biome (adaptation: Keeley *et al*., 2011; Lamont & He, 2017).

In the present paper we re‐evaluate published hypotheses on cypress biogeography, particularly the model for the whole family by Mao *et al*. (2012), as well as the hypothetical scenario of Crisp *et al*. (2011) that posits that multiple transoceanic disjunctions are the product of vicariance followed by differential extinction. Our main focus is the Southern Hemisphere subfamily Callitroideae using a much more comprehensive taxon sample and better area definitions than in previous studies. We use the first fully sampled molecular phylogeny of *Callitris* species to investigate the Cenozoic revival and spread of this genus across Australia and NC, including the role of adaptive shifts into fire‐dominated communities. Central to our approach is a re‐evaluation of the fossil record of the callitroids, focussing on the seed‐cone fossils of ‘*Callitris octothamna*’ (mid‐Cretaceous) and *Libocedrus mesibovii* (late Oligocene), which have not been used previously for calibration of molecular phylogenies. (The name of ‘*C. octothamna*’ is placed in quotes because it has not been formally published.) Fossils provide the only direct evidence for our hypothesis that differential extinction was responsible for at least some of the multiple transoceanic disjunctions in the extant genera (cf. Crisp *et al*., 2011). Fossils also provide direct evidence of turnover and rediversification in *Callitris*.

**Figure 1:**
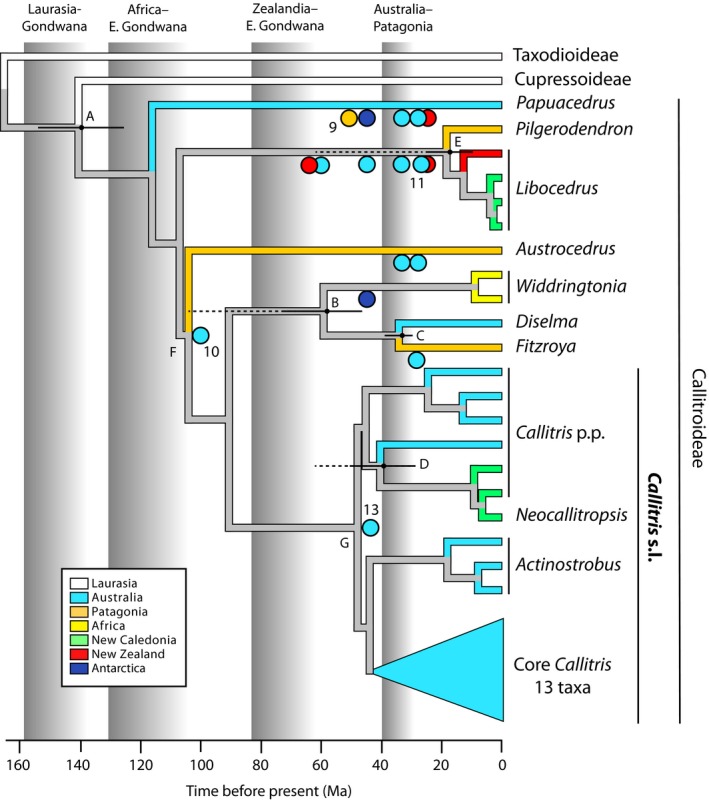
Simplified time tree of Cupressaceae estimated from the combined cpDNA + nDNA dataset, using a partitioned Beast analysis and calibrated using lognormal priors. Terminal branches and circles (indicating fossils) are coloured by geographical occurrence, as in the key. Fossils used to calibrate the Beast analysis are numbered as in Supporting Information Table S3 and Fig. S3. Fossil 10 (‘*Callitris octothamna*’) is at the position (node F) preferred by Bayes Factors. Fossil 11 (*Libocedrus mesibovii*) could be a *Pilgerodendron* (see text). Broad grey vertical bars show events in the breakup of Pangaea and Gondwana, graded from commencement to cessation of rifting. Black horizontal bars at nodes A to E show the 95% HPD of estimated divergence times (Table 2) between extant taxa in now‐separated parts of Laurasia and/or Gondwana. Dotted lines show gaps between these HPDs and the rifting event that putatively caused vicariance (B, D and E). Node G is the crown of *Callitris* s.l. The complete tree is shown in Fig. S11. Ma, million yr ago.

We test five hypotheses explaining transoceanic divergences (A to E in Fig. 1) by comparing our new molecular divergence‐time estimates with published tectonic rifting times. Mao *et al*. (2012) inferred four of these to be vicariance and one to be LDDE. Also, we test the fossil‐based extinction–rediversification hypothesis by examining lineages‐through‐time (LTT) plots for *Callitris* for signatures of these processes (Crisp & Cook, 2009), relative to its callitroid sister taxa, which suffered extinctions in Australia but did not rediversify there. Finally, we ask whether serotiny in *Callitris* could be an adaptation to fire by testing the expectation that the shift to a fire‐prone biome predated the origin of cone morphology associated with serotiny.

## Materials and Methods

### Taxon sampling

The taxon sample for the phylogeny (Supporting Information Table S1) comprised all 23 extant species of *Callitris* based on the taxonomy of Hill (1998), with the addition of the four species previously recognized as *Actinostrobus* (Western Australia) and *Neocallitropsis* (NC). Additionally, all three subspecies of *C. oblonga* (Hill, 1998) were sampled. To test the monophyly of *Callitris* s.l. and to estimate a well‐calibrated time‐tree, we also sampled 36 taxa across the rest of the family Cupressaceae, including all genera and most species of subfamily Callitroideae. We included two samples per species from *Callitris* and, where possible, the rest of Callitroideae. We could not obtain data for two of the four *Widdringtonia* species and one of the five *Libocedrus* species.

### DNA extraction, sequencing and alignment

Genomic DNA was extracted from *c*. 1.0 cm of dried foliage tissue using a slightly modified hexadecyltrimethylammonium bromide (CTAB) method (Murray & Thompson, 1980; Doyle, 1991). We sequenced *c*. 6 kb of standard loci from the chloroplast genome, including gene‐coding regions *rbcL*,* matK* and trnL‐trnF, as well as noncoding regions. We also sequenced three nuclear loci (c257 = inorganic pyrophosphatase; c22306 = erd1 and c36749 = alpha‐glucan protein synthase) using primers designed from an EST library of *C. intratropica* (Sakaguchi *et al*., 2011). Details of all loci and primer sequences, including references, are in Table S2. GenBank accession numbers for sequences are in Table S1. PCR amplification of all loci was carried out following the standard protocol from the Qiagen multiplex PCR kit (Qiagen, Hilden, Germany). PCR products were sequenced using both forward and reverse primers with an ABI prism bigdye terminator cycle‐sequencing ready‐reaction kit v.3.1 (Applied Biosystems, Waltham, MA, USA) and electrophoresed on an ABI prism 3100 genetic analyser. Sequences were edited using bioedit v.7.0.8.0 (Hall, 1999), aligned initially using mafft (Katoh & Toh, 2010) in cipres (Miller *et al*., 2010) and manually corrected in geneious ^®^ v.10.2.2, with exons adjusted to the open reading frame. Some hypervariable regions were excluded from the alignment and the final aligned lengths were: cpDNA = 6658, c257 = 282, c22306 = 729, c36759 = 273, total = 7942. The three nuclear loci could be sequenced successfully only within Callitroideae.

### Phylogenetic analysis

Maximum‐likelihood (ML) trees were generated using RaxML v.7.4.2 (Stamatakis *et al*., 2008) in cipres. DNA sequences were partitioned with substitution models comprising a SRD06 codon‐based model for the *rbcL* exon and separate general time‐reversible (GTR) + Γ models for the *matK* exon, the noncoding cpDNA and the three nuclear loci (c257, c22306 and c36749) combined into a single partition. Separate analyses of cpDNA (Fig. S1) and the three nuclear loci combined (Fig. S2) found no conflict between the two datasets in nodes with strong bootstrap support (BS > 80), so all loci were concatenated for subsequent analysis. The RaxML tree inferred from the combined data (Fig. S3) was then used as a starting tree for the Beast analyses.

Time‐calibrated phylogenies were inferred using Beast v.1.8.4 (Drummond *et al*., 2012) with an uncorrelated lognormal (UCLN) clock model (both Cupressaceae and *Callitris* datasets) and a random local clocks (RLC) model for *Callitris* only (Markov chain Monte Carlo (MCMC) chain stationarity could not be achieved using the RLC model with the full Cupressaceae dataset). Each partition (defined as for RaxML) was given a separate substitution and clock model in Beast. Relative fit of the alternative clock models (RLC and UCLN) in *Callitris* alone was evaluated using Bayes Factors (BF; Raftery, 1996) calculated from marginal likelihoods obtained by path (PS) and stepping‐stone (SS) sampling (Baele *et al*., 2012, 2013). This test indicated ‘very strong’ support (BF > 10) for the UCLN clock (Table S4), which was used in all subsequent Beast analyses. To model the speciation process, a birth–death model, corrected for incomplete sampling (Stadler, 2009), was used because species sampling was sparse in the non‐callitroid outgroups. To ensure stationarity and convergence of the Bayesian MCMC chains, three parallel chains were run and tracer v.1.6 (Rambaut *et al*., 2013) was used to check that the effective sample sizes in the combined logs (after a 10% burnin) were > 200 for all parameters. The MCMC chain length was set to a minimum of 100 million generations (full Cupressaceae dataset) and was increased to 200 million for clock model tests in *Callitris* to achieve stationarity and convergence. To display the results, the maximum clade credibility tree of *Callitris* was annotated in figtree v.1.4.3 (Rambaut, 2016), as recommended by the author.

### Fossil‐based calibration

For the time‐scaled (Beast) phylogenetic analysis of Cupressaceae, we used the fossil‐based calibration points listed in Table S3, including several from previous studies and some new ones, detailed in the next section. Calibration priors were given lognormal distributions, as recommended in theory (Morrison, 2008; Ho & Phillips, 2009). By contrast, Mao *et al*. (2012) used mostly uniform minimum‐age priors, whose flat distribution makes all ages equally probable between a hard minimum and maximum. But uniform priors increase temporal uncertainty in the calibrations and tend to bias estimates towards slower clock rates and older ages over the tree (Dornburg *et al*., 2012). A lognormal prior also sets a hard‐minimum age (i.e. the ‘offset’, which is usually set at the age of the fossil) but the lognormal tail limits regress of the age estimate into the past with diminishing probability (Ho & Phillips, 2009). In any case, we tested the fit of lognormal vs uniform prior distributions with otherwise matched datasets. Lognormal prior distributions were preferred very strongly by BF calculated from the Beast posterior marginal likelihoods (PS = 27.7, SS = 31.4; Table S4) and used in all subsequent Beast analyses.

### Assessment and model‐testing of previously unused fossils for calibration

Callitroideae has a rich fossil record (Fig. 1; Hill & Brodribb, 1999; Stockey *et al*., 2005; Wilf *et al*., 2009) and we evaluated some of its fossil taxa that have not been used previously for calibration. We chose these particular fossils because their age and phylogenetic placement potentially affected the biogeographical reconstruction of Callitroideae and *Callitris*. Fossils are typically fragmentary, limiting the number of characters available for their identification and phylogenetic assignment. One source of error has been the assignment of fossils to extant genera based on ancestral character states that are shared among multiple genera (symplesiomorphies), for example in *Libocedrus* (Whang & Hill, 1999). Therefore, to assess whether the fossils discussed below have unique defining characters (synapomorphies) for any lineages within Callitroideae, we scored potential characters (Table S5) and mapped them on the molecular phylogeny using mesquite v.3.4 (Maddison & Maddison, 2018) with parsimony and the ML model Mk1 (Lewis, 2001). Characters were scored for the fossil *C. leaensis* from the detailed description and photographic plate in Paull & Hill (2010).

1. ‘*Callitris octothamna*’ M.D. Peters (1985). These seed cone fossils are similar to cones of extant *Callitris* and potentially could greatly increase the age estimate of the genus, being from mid‐Cretaceous (99.6 Ma) sediments near Winton, Queensland. We assigned the calibration alternately to (a) the stem node of *Callitris*, being the most recent common ancestor (MRCA) of *Callitris*,* Fitzroya*,* Diselma* and *Widdringtonia* (hereafter, CFDW clade) and (b) its parent node. These assignments were based on mapping the characters phyllotaxis (Fig. S4), leaf dimorphism (Fig. S5) and cone‐scale arrangement (Fig. S6) on a tree. The fit of these calibrations relative to exclusion of the fossil, was assessed using BF.
2. *Libocedrus*: A number of *Libocedrus* fossils have been described from Australia and New Zealand (NZ) (Hill & Carpenter, 1989; Pole, 1998, 2007a,b; Whang & Hill, 1999) but none was used for calibration by Mao *et al*. (2012) even though they are relevant to biogeography. Extant *Libocedrus* is endemic to Zealandia (NC + NZ; Mortimer *et al*., 2017). We used the only known seed cone fossil, *L. mesibovii* (Hill & Carpenter, 1989), from the late Oligocene–early Miocene (*c*. 24 Ma), to calibrate the MRCA of sister genera *Libocedrus* and *Pilgerodendron*, and assessed its fit relative to its exclusion, using BF.

Assessment of these fossils is explained in more detail in Methods S1.

### Testing biogeographical hypotheses

Hypotheses proposed to explain transoceanic divergences between extant lineages of the callitroids (Crisp *et al*., 2011; Mao *et al*., 2012) were tested by comparing divergence times inferred from the molecular phylogeny with the timing of rifting events in the break‐up of Gondwana. The tests followed the protocol of Crisp *et al*. (2011: Box 2). This approach tests the prediction of a vicariance hypothesis that sister taxa on either side of a barrier should have diverged at the same time as the barrier originated. The test is two‐tailed, that is, vicariance is rejected if the divergence between the taxa either postdates or pre‐dates the origin of the barrier. The test also should take account of uncertainty in timing of both the lineage divergence times and the geological rifting. The test of vicariance is explicit because it addresses a specific divergence (node) in the phylogeny, which is hypothesized to be caused by the origin of a particular barrier. A rejection of a particular vicariance event does not generalize to a statement that vicariance does not explain the distribution of the whole taxon. It rejects only the specific hypothesis that ‘vicariance event *X* explains node *Y*’.

### Diversification of *Callitris*

The trend of *Callitris* lineages through the Cenozoic was plotted using mesquite. This plot was based on the Beast tree calibrated with the fossil ‘*Callitris octothamna*’ at the MRCA of *Austrocedrus* and *Callitris* (Fig. S7), with the addition of the fossil *C. leaensis*, which was inserted by hand mid‐way along the stem‐lineage of *Callitris* (cf. Figs S8, S9), diverging at 68 Ma and terminating at *c*. 34 Ma (= age of the fossil).

### Evolution of fire‐related traits

The seed‐cones of most *Callitris* species are woody and serotinous (Lamont *et al*., 1991; Ladd *et al*., 2013). That is, mature cones containing ripe seeds are held unopened in the canopy for one or more seasons, so that multiple generations of unopened cones are distributed along branches at any one time. Wildfires that burn tree canopies usually kill serotinous individuals, which then release their seeds that germinate in the ash‐bed and re‐establish the population. Serotiny is considered an adaptation to wildfire (Keeley *et al*., 2011; Lamont & He, 2017). Some species of *Callitris,* and most other callitroid genera, do not have serotinous cones but release their seeds as soon as they are ripe, generally within a year of pollination. If serotiny truly is an adaptation to wildfire (as opposed to an exaptation), it should have arisen by selection following the advent of recurrent fire into communities in which ancestral, nonserotinous species occurred – or the migration of an ancestral nonserotinous population into a new, fire‐prone habitat (Keeley *et al*., 2011; Lamont & He, 2017). This leads to the prediction that evolutionary transitions from nonserotiny to serotiny should correlate with transitions from nonfire‐prone to fire‐prone habitats, with the adaptive shift following the change of habitat, that is of the selective regime (Keeley *et al*., 2011). We tested this prediction using the data in Table S5 with the Pagel94 ML module (Pagel, 1994) in mesquite. All species of *Callitris* and many of the outgroups were visited in the field, where both traits were scored from direct observation. The remainder were scored from the literature, for example from Farjon (2005). Given the prediction above, the correlation model treated the serotiny variable (*Y*) as dependent upon the habitat flammability variable (*X*). As the Pagel94 model does not accept dimorphic traits, we scored alternative states for each of the duplicate tree‐tip samples in two dimorphic species (Table S5). Each trait was dimorphic in some species but no species was dimorphic for both traits; that is, duplicated tips were sufficient to score known combinations of the two traits.

## Results

### Phylogeny

**Figure 2:**
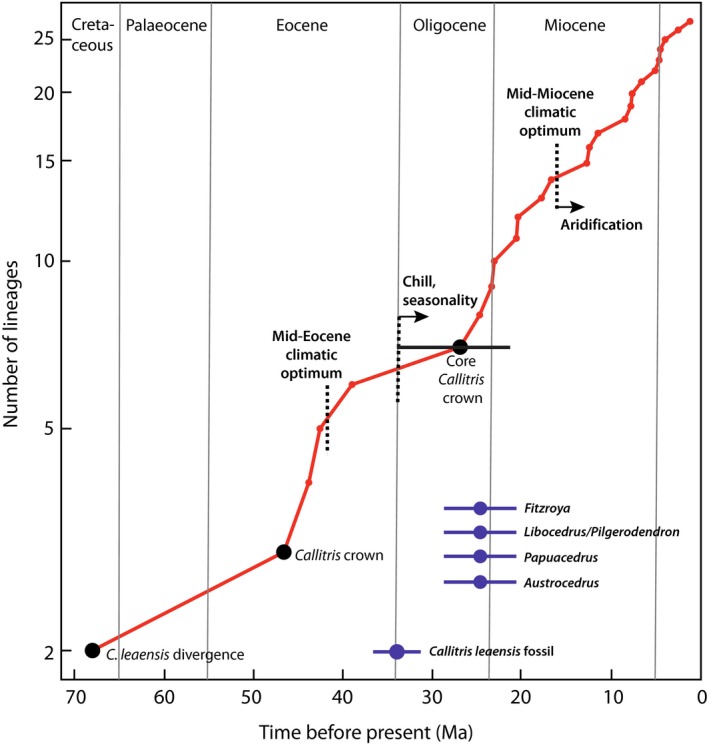
Lineages‐through‐time (LTT) plot for *Callitris*. Based on the Beast tree calibrated with the fossil ‘*Callitris octothamna*’ at the most recent common ancestor (MRCA) of *Austrocedrus* and *Callitris* (Supporting Information Fig. S7). The fossil *C. leaensis* was inserted by hand mid‐way along the stem‐lineage of *Callitris* (cf. Figs S8, S9), diverging at 68 million yr ago (Ma) and terminating at 34 Ma (= age of the fossil). Black filled circles are highlighted nodes from the tree. The core *Callitris* crown node marks the end of the plateau and likely extinction time, and the horizontal black bar is the 95% HPD of the age estimate for this node. Blue filled circles represent the most recent Australian fossils for callitroid genera, with bars representing uncertainty in their stratigraphic age. The *C. leaensis* fossil is not the most recent for the genus but clearly represents an ever‐wet habitat.

The Beast MCMC chains were stationary and converged after 200 million generations and the marginal likelihood tests very strongly preferred the UCLN clock model to the RLC model (BF: PS = 17.6, SS = 24.4; Table S4). Nonetheless, identical topologies were found from the combined data under both clocks and the age estimates were similar, with overlapping 95% HPDs (highest posterior densities). Hereafter, we use only the results from the preferred UCLN model. The RaxML (Figs S1–S3) and Beast (Figs S7, S10, S11) analyses of all Cupressaceae gave topologies consistent with Mao *et al*. (2012), except in the position of *Austrocedrus*, as noted below. Within *Callitris*, our phylogeny was more resolved, with higher nodal support, than that of Mao *et al*., who sampled only half (12) of the species. Hereafter the label ‘core *Callitris*’ (e.g. Figs 1, 2) is used for a clade that received maximum support (BS = 100 or PP = 1.0) in all trees. This clade includes 13 of the 23 species, and excludes some *Callitris* and all species previously assigned to other genera (*Actinostrobus* and *Neocallitropsis*, e.g. as in Farjon, 2005).

In the ML analyses, the cpDNA dataset (Fig. S1) placed *Austrocedrus* as sister to the CFDW clade without strong support (BS = 70). By contrast, the nuclear dataset (Fig. S2) placed it as sister to CFDW plus *Libocedrus* and *Pilgerodendron* (BS = 81). The combined data (Fig. S3) gave the same topology as cpDNA (BS = 72). Additionally, in trees from all three datasets, and in both ML and Beast analyses, the branch subtending *Austrocedrus* and its sister group was always very short. Given this uncertainty, the Beast analysis of the combined data was alternately constrained to give the nDNA topology (Fig. S10) and the result compared with the unconstrained analysis (Figs S7). Both topologies were compared in alternate downstream analyses where they could have made a difference to key results. However, the results were virtually identical in divergence times relevant to biogeography (Table 1), fossil trait reconstructions for calibration placement (Table 2), and mapping of fire‐related traits (not shown).

**Table 1:** Estimated divergence times from Beast for key nodes for testing biogeographic hypotheses

Nodes	Not calibrated *with* ‘*Callitris octothamna*’		‘*C. octothamna*’ (calibration 10) at parent of *Callitris* stem node		‘*C. octothamna*’ at *Callitris* stem node
	Unconstrained	Constrained	Unconstrained	Constrained	Unconstrained
A. Callitroideae–Cupressoideae	127.0 (105–148)	127.0 (106–150)	139.7 (125–154)	139.6 (126–156)	148.4 (134–163)
B. *Widdringtonia*–(*Diselma*,* Fitzroya*)	52.5 (41–66)	52.3 (41–66)	58.3 (45–73)	58.7 (45–73)	64.6 (47–82)
C. *Diselma*–*Fitzroya*	32.8 (30–38)	32.8 (30–38)	33.2 (30–39)	33.2 (31–39)	33.5 (30–41)
D. *Callitris* NC–*C*. *macleayana*	34.8 (25–46)	35.3 (25–46)	39.3 (28–50)	39.4 (28–51)	43.0 (31–56)
E. *Pilgerodendron*–*Libocedrus*	15.6 (9–24)	15.7 (9–24)	17.3 (10–26)	17.3 (10–27)	18.5 (11–28)
F. CFDW stem	87.2 (71–105)	87.6 (71–105)	103.0 (100–110)	103.1 (100–110)	113.0 (106–112)
G. *Callitris* crown	41.6 (33–51)	42.0 (33–52)	46.9 (38–57)	47.3 (38–57)	51.5 (41–62)

Node labels (A–G) refer to Fig. 1. Primary columns represent different calibration settings for ‘*Callitris octothamna*’, including its exclusion. Sub‐columns differ in whether monophyly of the CFDW–*Libodedrus*–*Pilgerodendron* clade was constrained, to the exclusion of *Austrocedrus*. All these results are from the combined cpDNA + nDNA dataset. The unconstrained topology reflects that from cpDNA alone and places *Libocedrus* + *Pilgerodendron* as sister to CFDW (Supporting Information Fig. S1). The constrained topology reflects that from nDNA alone (Fig. S2). Values are median age estimates in million yr ago (Ma) (95% HPD). NC, New Caledonia; CFDW, *Callitris*,* Fitzroya*,* Diselma* and *Widdringtonia*.

**Table 2:** Mapping of fossil characters to guide calibration placement on the molecular phylogeny: proportional likelihoods at candidate nodes

Characters and states	Tree: *Austrocedrus* in			Tree: *Austrocedrus* out		
	Node B (CFDW stem)	Node A (*Callitris* stem)	*Callitris* crown	Node B (CFDW stem)	Node A (*Callitris* stem)	*Callitris* crown
Phyllotaxis ternate	0.03	0.51*	1.00*	0.03	0.33*	1.00*
Phyllotaxis decussate	0.96*	0.48*	0.00	0.97*	0.65*	0.00
Leaves monomorphic	0.16	0.85	1.00*	0.20	0.85	1.00*
Leaves dimorphic	0.83	0.14	0.00	0.80	0.15	0.00
Cone‐scales two whorls of three	0.02	0.26*	1.00*	0.01	0.16*	1.00*
Cone‐scales decussate	0.98*	0.74*	0.00	0.99*	0.84*	0.00

Proportional likelihoods under the Mk1 model (Lewis, 2001) were sampled at candidate nodes on alternative topologies: (a) with *Austrocedrus* sister to the CFDW clade (‘*Austrocedrus* in’), as supported by cpDNA and the combined data, and (b) with *Libocedrus* + *Pilgerodendron* constrained to be sister to CFDW (‘*Austrocedrus* out’), as supported by the nDNA alone. For each character, the plesiomorphic (ancestral) state is listed after the derived state. The transition between the plesiomorphic and derived state for each character is reconstructed as occurring between node B and the *Callitris* crown node, possibly in the *Callitris* stem (node A). The node labels refer to Supporting Information Figs S4–S6. CFDW, *Callitris*,* Fitzroya*,* Diselma* and *Widdringtonia*.

*States judged ‘best’ (likelihoods are not significantly different at that node if both states are asterisked).

### Assessment and model‐testing of previously unused fossils for calibration

Three models were compared with respect to the assignment of ‘*Callitris octothamna*’ as a calibration and ranked by their marginal Log_e_ likelihoods (Table S4). The first‐ranked model, with ‘*C. octothamna*’ placed at the MRCA of *Callitris* and *Austrocedrus*, was positively preferred (BF: PS = 4.7, SS = 5.2) to the third‐placed model, with ‘*C. octhothamna*’ placed at the *Callitris* stem node. The second‐ranked model (‘*C. octothamna*’ calibration omitted) differed minimally from the first‐ranked model (BF: PS = 0.84, SS = 0.22; Table S4), at a level considered by Raftery (1996) as ‘not worth more than a bare mention’ (Raftery, 1996). Consequently, both best models were used in downstream analyses. Divergence times (Table 1) estimated using the best model, with placement of ‘*C. octothamna*’ at the CFDW stem (Fig. S7), were older than those estimated without this calibration (Fig. S11), although the 95% HPDs overlapped. The largest difference (15.9 Ma) was at the calibrated node itself.

Exclusion of the calibration using *Libocedrus mesibovii* was very strongly preferred to its inclusion (BF: PS = 33, SS = 600; Table S4), so it was thereafter omitted.

### Testing biogeographical hypotheses

The divergence between the Cupressoideae and Callitroideae (node A in Fig. 1) is consistent with vicariance (Fig. 1) under all calibration models (Table 1). The 95% HPDs of the divergence‐time estimates overlap the period (*c*. 160–140 Ma) when Laurasia and Gondwana finally separated as Pangaea broke up (McLoughlin, 2001). Likewise, all five divergence‐time estimates for *Diselma* and *Fitzroya* are consistent with the separation of Australia, South America and Antarctica *c*. 40–34 Ma (Fig. 1; Table 1). All other divergences (B, D, E) relevant to biogeography are too recent to be explained by vicariance because the upper (older) 95% HPDs are > 10 Ma younger than the completion of rifting between the respective landmasses (Fig. 1; Table 1). These are: B, between *Widdringtonia* in West Gondwana (Africa) and *Diselma* + *Fitzroya* in East Gondwana (*c*. 130–105 Ma); D, between *Callitris* in NC and the *C. baileyi* clade in Australia (*c*. 80–65 Ma); and E, between *Pilgerodendron* (Patagonia) and *Libocedrus* in Australia and NZ (*c*. 80–65 Ma). Details of biogeographical hypothesis tests for all callitroid genera follow.

*Papuacedrus* was once extant in South America, Antarctica, southern Australia and NZ (Fig. 1; Wilf *et al*., 2009) but is now extinct in all those places. According to the reconstruction by Mao *et al*., its range expanded from South America to area U (Australasia, including New Guinea) between 130 and 52 Ma, followed by vicariance separating the South American fossil from the extant New Guinea lineage. This inference was based on the fossil record alone because, with a single living species in New Guinea, there is no molecular divergence time against which this hypothesis can be tested.

*Libocedrus* and possibly also *Pilgerodendron* (see Methods S1) have an extensive fossil record between 60 and 22 Ma, exclusively from Australia and NZ (Fig. 1). Mao *et al*. (their Fig. S4) postulate that the ancestor of *Libocedrus* + *Pilgerodendron* spread from South America to Australasia (their area U) after *c*. 105 Ma and then *c*. 35 Ma, diverged by vicariance into these two genera. However, the latter event is much too young to be explained by the tectonic separation of Zealandia and South America (85–65 Ma). Our most conservative dating (Fig. 1; Table 1, column 5) of the divergence between these genera was 11–28 Ma, supporting a hypothesis of trans‐Pacific dispersal. The dispersal could have gone in either direction but, given the extensive fossil record for the clade in Australasia during that period (reviewed in Wilf *et al*., 2009), perhaps it was the source. There is no known fossil record of either genus from South America (Wilf *et al*., 2009).

*Austrocedrus* fossils are known from Australia at the right time (36–30 Ma) for the genus to be vicariant in South America and Australia, followed by extinction of the Australian population. Consistently with this, Mao *et al*. estimated the divergence at *c*. 30 Ma.

We estimate that *Widdringtonia* (southern Africa) diverged from *Diselma* + *Fitzroya* (East Gondwana) 73–41 Ma (Fig. 1; Table 1), long after these land masses finished separating (*c*. 105 Ma; McLoughlin, 2001), so this appears to have been a transoceanic dispersal. Mao *et al*. (2012) also reconstructed a dispersal to Africa around the Cretaceous–Palaeogene boundary but from a source in South America. However, fossil wood has been tentatively identified as *Widdringtonia* from Miocene to Quaternary sediments on Kerguelen Island, which lies in the southern Indian Ocean (= *Widdringtonioxylon antarcticum*; Philippe *et al*., 1998). This small island is part of the much more extensive Kerguelen Plateau, which is mostly submerged now but supported terrestrial flora in the early Late Cretaceous (McLoughlin, 2001). Therefore, Kerguelen could have been a stepping stone if the ancestor of *Widdringtonia* actually dispersed from Antarctica (when part of East Gondwana) to Africa.

*Fitzroya* has a single species endemic to Patagonia and is sister to the monotypic *Diselma* in Tasmania. Mao *et al*. reconstructed the MRCA of these genera as present in East Gondwana from *c*. 110 Ma, then diverging (nonvicariant, i.e. within East Gondwana) *c*. 45 Ma, followed by vicariant speciation within *Fitzroya c*. 35 Ma when Australia, Antarctica and South America rifted apart. This was followed by extinction of the Australian *Fitzroya* population. Our dating linked the *Fitzroya*–*Diselma* divergence to that rifting event, but given the error bars (Fig. 1), either that or the divergence between Australia and South America within *Fitzroya* could be related to it – but not both.

Multiple genera were more widespread in the past and all callitroid genera except *Widdringtonia* are represented in Australia by either fossils or living species (Fig. 1). The relevance of fossils to tests of biogeographical hypotheses is explained under ‘Fossils combined with molecular evidence reveal the extent of turnover’ in the Discussion section.

### Diversification and substitution rates

The log‐linear plot (Fig. 2) depicting the accumulation of *Callitris* lineages‐through‐time (LTT) in the ‘*C. octothamna*’‐calibrated tree rises steeply until it plateaus between *c*. 40 and 28 Ma, possibly indicating an extinction event in the late Oligocene (Crisp & Cook, 2009). Thereafter, the LTT graph rises steeply and linearly to the present, indicating a more or less constant rate of diversification. The estimated ages of the last known Australian fossils of at least four callitroid genera overlap the plateau in the *Callitris* LTT plot (Fig. 2). The alternative tree with no ‘*C. octothamna*’ calibration gave a very similar plot (not shown), shifted *c*. 5 Ma towards the present.

### Evolution of fire‐related traits

A significant correlation (*P *=* *0.04) in which serotiny (*Y*) depends upon habitat flammability (*X*) was found using Pagel's (1994) test of correlated (discrete‐state) character evolution from 100 simulations on the tree (Figs S8, S9). Using the alternative tree with no ‘*C. octothamna*’ calibration (Fig. S11), the correlation also was significant (*P *=* *0.01).

Mapping these traits onto the phylogeny indicated a possible time‐lag between transitions, with the habitat shifts occurring before the origin of serotiny, at least within *Callitris*. In parsimony mapping, the MRCA of all *Callitris* was reconstructed as having a fire‐prone habitat (Fig. S9), whereas the same node was reconstructed indecisively – as either serotinous or not (Fig. S8). Similarly, ML mapping indicated decisively that the transition to fire‐prone habitat had occurred by the crown node of *Callitris* (proportional likelihood ≥ 0.90*, which is significant) on both trees (with and without calibration using ‘*C. octothamna*’) (Table 3). By contrast, the proportional likelihood of serotiny did not exceed the decisive value of 0.90 until two nodes later (core *Callitris* crown node). These traits are homoplasious, with multiple gains and/or losses in both the Cupressoideae (*Cupressus*,* Juniperus* and *Tetraclinis*) and the Callitroideae (*Callitris* and *Widdringtonia*) (Figs S8, S9). Within *Callitris*, absences of serotiny reconstructed as reversals in the MRCA of the *C. macleayana*‐NC clade and the MRCA of the *C. columellaris s.l*. clade*. Callitris baileyi* also lacks serotiny but the trait reconstruction was equivocal, that is serotiny could have been lost in *C. baileyi* or it could have originated independently in its sister species (*C. roei* and *C. drummondii*).

**Table 3:** Fire‐related trait transitions: proportional likelihoods at nodes

Node	Tree with ‘*C. octothamna*’ calibration at CFDW stem node			Tree with no ‘*C. octothamna*’ calibration		
	Age (Ma)	*X* = habitat flammability	*Y* = cone serotiny	Age (Ma)	*X* = habitat flammability	*Y* = cone serotiny
*Widdringtonia* – *Callitris*	90	0.52	0.40	76	0.51	0.38
*Callitris* crown – *C. leaensis*	72	0.57	0.44	59	0.56	0.40
*Callitris* crown (= *C. roei* – *C. oblonga*)	47	0.90*	0.70	42	0.90*	0.68
*Actinostrobus* – core *Callitris* clade	43	0.92*	0.82	39	0.92*	0.81
Core *Callitris* crown (= *C. canescens* – *C. oblonga*)	27	0.99*	0.99*	24	0.99	0.99*

Proportional likelihoods under the Mk1 model (Lewis, 2001) were sampled at successive nodes (top row is nearest the root). Alternative Beast trees differ in node ages, depending on whether they were calibrated with ‘*C. octothamna*’. As the characters are binary, only the proportional likelihood for the derived state is shown. Correlations between these traits, where *Y* depends upon *X*, were assessed using the Pagel94 test. The node references are to the most recent common ancestor (MRCA) of the named taxa (see Supporting Information Figs S8, S9). Ages are median estimates for that node. CFDW, *Callitris*,* Fitzroya*,* Diselma* and *Widdringtonia*.

*States judged ‘best’ (likelihoods are not significantly different at that node if both states are asterisked). Ma, million yr ago.

Recently, serotiny was reported in *Protodammara*, a putative Cupressaceae fossil from mid‐Cretaceous NZ (Mays *et al*., 2017). The authors suggest an affinity to members of Cupressaceae that are not closely related to Callitroideae, so serotiny is likely to be independently evolved in *Protodammara* and the callitroids. The reference by Mays *et al*. to serotiny in a mid‐Cretaceous fossil of *Widdringtonia* (McIver, 2001) is based on a likely misidentification of this fossil (Stockey *et al*., 2005; Crisp & Cook, 2011).

## Discussion

### Biogeography: post‐Gondwanan transoceanic dispersals

The callitroid cypresses have an ancient fossil record, tracing back to the separation of Gondwana and Laurasia > 150 Ma (Hill & Brodribb, 1999; Wilf *et al*., 2009). Using the most comprehensive phylogeny of the callitroids (Fig. 1), we have confirmed that they spread widely across Gondwana before it broke up (Crisp *et al*., 2011; Leslie *et al*., 2012; Mao *et al*., 2012). At least some divergences are consistent in timing and location with vicariance hypotheses, notably Cupressoideae–Callitroideae (Laurasia–Gondwana) and *Diselma*–*Fitzroya* (Australia–South America), because the 95% HPD intervals of their divergence‐time estimates overlap the relevant tectonic rifting periods (Fig. 1; Table 1).

By contrast, our tests of biogeographical hypotheses found three post‐Gondwanan transoceanic divergences that were too young to be explained by vicariance, even from our oldest estimates (Table 1, last column), and therefore likely resulted from long‐distance dispersal and establishment. These were the divergences between (a) *Pilgerodendron* (Patagonia) and *Libocedrus* (Zealandia), (b) *Callitris macleayana* (Australia) and the NC clade, and (c) *Widdringtonia* (southern Africa) and *Diselma* (Tasmania) + *Fitzroya* (Patagonia). Of these, (a) and (b) were not inferred as transoceanic dispersals by Mao *et al*. (2012) because those authors lumped Zealandia into Australia (their area ‘U’), yet Zealandia rifted away from Australia (then part of East Gondwana) in the Cretaceous, long before the trans‐Pacific divergences in these two clades (Fig. 1; Table 1). By treating Australia and Zealandia as separate regions, we have been able to more accurately reconstruct their biogeography.

In a large modelling analysis across all conifers, Condamine *et al*. (2017) concluded that islands, especially those of Zealandia, were refugia and centres of diversification, from where they dispersed back to continents such as Australia. The authors inferred that conifer diversification was faster on islands than on continents, due to lower speciation and higher extinction rates in the latter. This is not supported by our data for the callitroids, except that there was extensive extinction in Australia during the Cenozoic (Figs 1, 2). Both callitroid clades in Zealandia appear to have dispersed across the Tasman Sea from Australia during the Neogene, with no evidence for dispersal in the opposite direction. Within Australia, *Callitris* diversified rapidly into multiple habitats following cooling and aridification (Fig. 2). Today, more than two‐thirds of the callitroids (25 of 38 spp.) are restricted to Australia but all are *Callitris* species, except *Diselma archeri*. Condamine *et al*. sampled barely half the callitroid species and most of the missing taxa are Australian *Callitris* (14 of 24 spp.). Therefore, their analysis would have underestimated the extent and speed of the Australian (i.e. continental) radiation.

### Fossils combined with molecular evidence reveal the extent of turnover

The present‐day distribution of callitroids reflects long persistence in the southern continents, but the biogeographical history is obscured by extensive extinctions following global climatic change at the end of the Eocene, especially in Australia (Hill, 2004; Hill & Brodribb, 2006). Fossils of many extant genera have been found in places where they no longer exist, especially in Australia (Figs 1, 2). This is not surprising because, in general, extinct species vastly outnumber living species, even if only the Cenozoic period is considered (Marshall, 2017). How does knowledge of extinctions change the historical interpretation of the biogeographical pattern of extant callitroids (Crisp *et al*., 2011)? Our critical examination of significant but hitherto neglected fossils has revealed more extensive past distributions than used previously for biogeography, and these suggested alternative biogeographical reconstructions, for example *Pilgerodendron* and *Widdringtonia*.

Some fossils identified as *Fitzroya* from late Oligocene to Miocene sediments in Tasmania (Hill & Paull, 2003; Paull & Hill, 2010) appear to postdate the inferred vicariant divergence between *Diselma* in Australia and the single extant species of *Fitzroya* in Patagonia (Table 1). Did *Fitzroya* diverge from *Diselma* sympatrically in Tasmania and then go extinct there, or simply differentiate into *Diselma*? The latter possibility seems unlikely because the oldest (Early Oligocene) *Fitzroya* fossils, from the Lea River in Tasmania, include a female cone in which the cone‐scale phyllotaxis is ternate (Paull & Hill, 2010). This character state is derived relative to the decussate cone‐scale phyllotaxis of *Diselma* (Fig. S9).

A similar conundrum is the possible co‐occurrence of *Pilgerodendron* (= *Libocedrus mesibovii*, fossil 11 in Fig. 1) with *Libocedrus jacksonii* in Tasmania in the late Oligocene (*c*. 25 Ma) (Hill & Carpenter, 1989), about the same time as the trans‐Pacific divergence between these genera (10–26 Ma; column 3 in Table 1). We suggest that *L. mesibovii* could be a *Pilgerodendron* because it shares a putative synapomorphy (asymmetric cones; see Methods S1) with the extant Patagonian population. All other fossils assigned to *Libocedrus* are vegetative only, so they might have had asymmetric cones before this feature was lost after the extant lineage established in Zealandia.

*Callitris* fossils are unknown outside Australia, and even there the record is minimal (Peters, 1985; Hill & Brodribb, 1999; Paull & Hill, 2010). ‘*Callitris octothamna*’ (Peters, 1985) is by far the oldest known callitroid fossil (mid‐Cretaceous) and so is important for molecular clock calibration. However, our phylogenetic mapping of morphological traits (Methods S1) indicates that it was not a *Callitris* but more likely an early member of the Callitroideae*. Callitris leaensis*, which is represented by a good range of leaf material, clearly shows features such as exposed stomata scattered over the abaxial surface, which characterize extant species from ever‐wet habitats (Paull & Hill, 2010). The age of this fossil coincides with the global chilling and cooling triggered by the opening of the Southern Ocean (Zachos *et al*., 2001). This led to contraction of the Eocene rainforests and expansion of seasonally dry, fire‐dominated sclerophyll communities Australia‐wide (Hill, 2004; Hill & Brodribb, 2006; Crisp & Cook, 2013). A plateau in the Lineages‐through‐time plot (Fig. 2) indicates that *Callitris* suffered a pulse of extinction at this time and then rediversified through the Neogene and until the present. The same period marks the last known occurrence of four (or five) other callitroid genera in Australia (Fig. 2). That is, ever‐wet taxa went extinct as their habitats disappeared, but *Callitris* rediversified because some species adapted and moved into dry, fire‐prone biomes.

### Fire‐related adaptation

Serotiny is a trait hypothesized to enable a population of fire‐sensitive woody plants to survive despite being killed by recurrent crown fires. It is found in communities with intermediate fire frequency, relative to the generation time of the plants. If fire recurrence is very infrequent (e.g. in rainforest), there is no effective selection for serotiny, and if recurrence is so frequent that a population cannot re‐establish between fires (e.g. in savannah), it will go extinct (Lamont & Enright, 2000; Ladd *et al*., 2013) unless there are adaptations to resist (thick bark) or vegetatively recover (stem or basal sprouts) from fire (Bowman *et al*., 2017). Our correlation tests indicate that serotiny in callitroids and cupressoids has a statistically significant dependence upon the putative selective agent, habitat flammability. Note that Pagel's (1994) test does not rely upon reconstruction of ancestral states. Although correlation alone cannot indicate causation, this result is consistent with the hypothesis that shifts to serotiny in *Callitris* are adaptations resulting from shifts into a crown‐fire regime (Keeley *et al*., 2011).

However, ancestral state reconstruction indicated that serotinous cones originated up to 20 Ma later than the shifts from nonfire‐prone to fire‐prone forest and scrub habitats (Table 3; Figs S8, S9). This time‐lag suggests that serotiny was not the trait enabling survival of *Callitris* in fire‐prone communities, at least initially. Alternative traits, such as those described below, might have enabled survival under wildfire. Such traits would be considered exaptations if they were ancestral, having been selected by factors other than wildfire (Bradshaw *et al*., 2011). Serotiny, originating later, might have been an adaptation resulting from a change in fire regime, for example towards crown fires with greater intensity, as climatic drying increased later in the Oligocene.

Two groups within *Callitris* lack serotiny. One is the clade including *C. columellaris*,* C. intratropica* and *C. glaucophylla* and reconstructs as having lost serotiny secondarily (Fig. S8). These three species inhabit sclerophyll or savannah communities subject to frequent surface fires, rather than crown fires. They release their seeds as soon as they mature and depend upon the soil seed‐bank for regeneration (Lamont *et al*., 1991). Moreover, these species are community dominants, forming extensive stands, and it has been suggested that they survive partly by suppressing low‐intensity fire where the stands are sufficiently dense, and partly in fire refugia such as rocky areas or patches that are unburned by chance (Cohn *et al*., 2011; Trauernicht *et al*., 2012). They have thick bark and even seedlings can withstand surface fires with low flame heights (Bowman *et al*., 2017). These traits are widely present in nonflammable communities and thus are likely exaptations for fire.

The second nonserotinous group occurs in wet habitats that burn infrequently (*C. baileyi*,* C. macleayana* and the three NC species). They belong to a clade that also includes *C. roei* and *C. drummondii*, both of which are serotinous and occur in communities prone to recurrent crown fires. The most recent common ancestor (MRCA) of the inclusive clade reconstructs as having a flammable habitat (Fig. S9) but could have been either serotinous or not (Fig. S8). If it was serotinous, then serotiny was lost in *C. baileyi* and the *C. macleayana*‐NC clade. If the MRCA was not serotinous, then serotiny was gained in *C. roei* and *C. drummondii*.

### Conclusion

The Gondwanan subfamily Callitroideae of the cypresses has a long history (150 Ma) in Australia: five of the eight extant genera were present in ever‐wet rainforests by the early Oligocene. However, global climate change through the Oligocene resulted in contraction of rainforest and expansion of sclerophyll communities, and all the genera except *Callitris* were extinguished in Australia by the mid‐Miocene. *Callitris* survived and rediversified through to the present by adaptively shifting into seasonally dry environments, some of which were prone to crown fires, and where serotiny might have been a selective advantage. We have shown how fossils can be integrated with molecular phylogenies to illuminate how species turnover and renewal have shaped the biogeography of southern cypresses.

## Author contributions

MDC led the project including design of the research, data analysis, interpretation of results and writing the manuscript; the project was originally conceived by DMJSB, LGC, MDC and YI, and LGC and MC were involved in design of the research; all authors contributed to the field sampling; SS led the laboratory work, including loci selection, sequencing and sequence assembly and alignment; LGC and DMJSB contributed to interpretation of results and manuscript writing.

## Supporting information

Please note: Wiley Blackwell are not responsible for the content or functionality of any Supporting Information supplied by the authors. Any queries (other than missing material) should be directed to the *New Phytologist* Central Office.

**Fig. S1** Maximum‐likelihood phylogeny of the cpDNA dataset, estimated using a partitioned RaxML analysis.
**Fig. S2** Maximum‐likelihood phylogeny of the nDNA dataset, estimated using an unpartitioned RaxML analysis.
**Fig. S3** Maximum‐likelihood phylogeny of combined cpDNA and nDNA datasets, using a partitioned RaxML analysis.
**Fig. S4** Leaf phyllotaxis reconstructed on the tree, to guide placement of ‘*C. octothamna*’ fossil for calibration.
**Fig. S5** Leaf dimorphism and monomorphism reconstructed on the tree, to guide placement of ‘*C. octothamna*’ fossil for calibration.
**Fig. S6** Cone‐scale phyllotaxis reconstructed on the tree, to guide placement of ‘*C. octothamna*’ fossil for calibration.
**Fig. S7** Time tree of Cupressaceae estimated from the combined cpDNA‐nDNA dataset, calibrated with ‘*C. octothamna*’ but not constrained to nDNA topology.
**Fig. S8** Parsimony reconstruction of the fire‐adaptive trait cone serotiny.
**Fig. S9** Parsimony reconstruction of fire‐prone habitat.
**Fig. S10** Time tree of Cupressaceae estimated in Beast from the combined cpDNA‐nDNA dataset, calibrated with ‘*C. octothamna*’ and constrained to nDNA topology.
**Fig. S11** Time tree of Cupressaceae estimated from the combined cpDNA‐nDNA dataset, not calibrated with ‘*C. octothamna*’ and not constrained to nDNA topology.
**Methods S1** Further details on assessment of previously unused fossils for calibration.
**Notes S1** References for Supporting Information.
**Table S1** Taxa sampled, sample sources, their geographic origin and GenBank accession numbers for sequences included in this study.
**Table S2** Loci sequenced, primers used for PCR and their design sources.
**Table S3** Molecular‐clock calibrations.
**Table S4** Model comparisons using Bayes factors calculated from marginal likelihoods in Beast.
**Table S5** Trait data: fossil morphology and fire‐adaptive traits.
