# Supplementary material for: Turnover of southern cypresses in the post‐Gondwanan world: extinction, transoceanic dispersal, adaptation and rediversification
Source: New Phytol. 2018 Nov 23;221(4):2308–19. doi: 10.1111/nph.15561 (PMC6587739; doi:10.1111/nph.15561)
Supplement: Supplementary file 1 — Fig. S1 Maximum‐likelihood phylogeny of the cpDNA dataset, estimated using a partitioned RaxML analysis. Fig. S2 Maximum‐likelihood phylogeny of the nDNA dataset, estimated using an unpartitioned RaxML analysis. Fig. S3 Maximum‐likelihood phylogeny of combined cpDNA and nDNA datasets, using a partitioned RaxML analysis. Fig. S4 Leaf phyllotaxis reconstructed on the tree, to guide placement of ‘C. octothamna’ fossil for calibration. Fig. S5 Leaf dimorphism and monomorphism reconstructed on the tree, to guide placement of ‘C. octothamna’ fossil for calibration. Fig. S6 Cone‐scale phyllotaxis reconstructed on the tree, to guide placement of ‘C. octothamna’ fossil for calibration. Fig. S7 Time tree of Cupressaceae estimated from the combined cpDNA‐nDNA dataset, calibrated with ‘C. octothamna’ but not constrained to nDNA topology. Fig. S8 Parsimony reconstruction of the fire‐adaptive trait cone serotiny. Fig. S9 Parsimony reconstruction of fire‐prone habitat. Fig. S10 Time tree of Cupressaceae estimated in Beast from the combined cpDNA‐nDNA dataset, calibrated with ‘C. octothamna’ and constrained to nDNA topology. Fig. S11 Time tree of Cupressaceae estimated from the combined cpDNA‐nDNA dataset, not calibrated with ‘C. octothamna’ and not constrained to nDNA topology. Methods S1 Further details on assessment of previously unused fossils for calibration. Notes S1 References for Supporting Information. Table S1 Taxa sampled, sample sources, their geographic origin and GenBank accession numbers for sequences included in this study. Table S2 Loci sequenced, primers used for PCR and their design sources. Table S3 Molecular‐clock calibrations. Table S4 Model comparisons using Bayes factors calculated from marginal likelihoods in Beast. Table S5 Trait data: fossil morphology and fire‐adaptive traits. [file NPH-221-2308-s001.pdf]

New Phytologist

***New Phytologist* Supporting Information**

Article title: **Turnover of southern cypresses in the post-Gondwanan world: extinction, transoceanic dispersal, adaptation and rediversification**

Authors: Michael D. Crisp, Lyn G. Cook, David M. J. S. Bowman, Meredith Cosgrove, Yuji Isagi and Shota Sakaguchi

Article acceptance date: 15 October 2018

The following Supporting Information is available for this article:

Fig. S1 Maximum likelihood phylogeny of the cpDNA dataset, estimated using a partitioned RAxML analysis.

Fig. S2 Maximum likelihood phylogeny of the nDNA dataset, estimated using an unpartitioned RAxML analysis.

Fig. S3 Maximum likelihood phylogeny of combined cpDNA and nDNA datasets, using a partitioned RAxML analysis.

Fig. S4 Leaf phyllotaxis reconstructed on the tree to guide placement of *"C. octothamna"* fossil for calibration.

Fig. S5 Leaf dimorphism and monomorphism reconstructed on the tree, to guide placement of *"C. octothamna"* fossil for calibration.

Fig. S6 Cone-scale phyllotaxis reconstructed on the tree, to guide placement of *"C. octothamna"* fossil for calibration.

Fig. S7 Time tree of Cupressaceae estimated from the combined cpDNA-nDNA dataset, calibrated with *"C. octothamna"* but not constrained to nDNA topology.

Fig. S8 Parsimony reconstruction of the fire-adaptive trait cone serotiny.

Fig. S9 Parsimony reconstruction of fire-prone habitat.

Fig. S10 Time tree of Cupressaceae estimated in BEAST from the combined cpDNA-nDNA dataset, calibrated with *"C. octothamna"* and constrained to nDNA topology.

Fig. S11 Time tree of Cupressaceae estimated from the combined cpDNA-nDNA dataset, not calibrated with *"C. octothamna"* and not constrained to nDNA topology.

Table S1 Taxa sampled, sample sources, their geographic origin and GenBank accession numbers for all sequences included in this study.

Table S2 Loci sequenced, primers used for PCR and their design sources.

Table S3 Molecular-clock calibrations.

Table S4 Model comparisons using Bayes factors calculated from marginal likelihoods in BEAST.

Table S5 Trait data: fossil morphology and fire-adaptive traits.

Methods S1 Further details on assessment of previously unused fossils for calibration.

Notes S1 References for Supporting Information.

Fig. S1 Maximum likelihood phylogeny of the cpDNA dataset, estimated using a partitioned RAxML analysis. Bootstrap values ≥ 70 are shown at nodes.

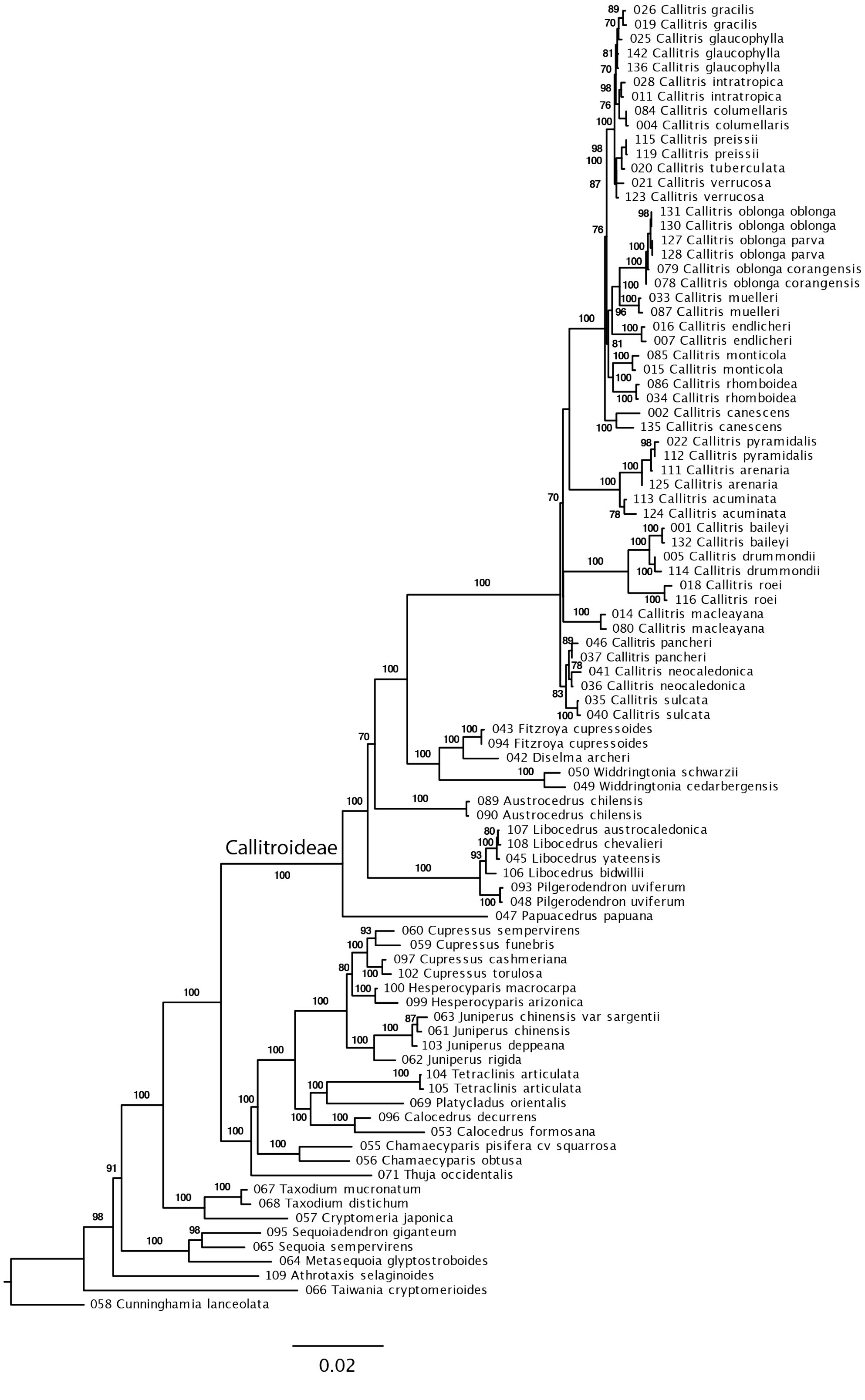
026 Callitris gracilis
019 Callitris gracilis
025 Callitris glaucophylla
142 Callitris glaucophylla
136 Callitris glaucophylla
028 Callitris intratropica
011 Callitris intratropica
084 Callitris columellaris
004 Callitris columellaris
115 Callitris preissii
119 Callitris preissii
020 Callitris tuberculata
021 Callitris verrucosa
123 Callitris verrucosa
131 Callitris oblonga oblonga
130 Callitris oblonga oblonga
127 Callitris oblonga parva
128 Callitris oblonga parva
079 Callitris oblonga corangensis
078 Callitris oblonga corangensis
033 Callitris muelleri
087 Callitris muelleri
016 Callitris endlicheri
007 Callitris endlicheri
085 Callitris monticola
015 Callitris monticola
086 Callitris rhomboidea
034 Callitris rhomboidea
002 Callitris canescens
135 Callitris canescens
022 Callitris pyramidalis
112 Callitris pyramidalis
111 Callitris arenaria
125 Callitris arenaria
113 Callitris acuminata
124 Callitris acuminata
001 Callitris baileyi
132 Callitris baileyi
005 Callitris drummondii
114 Callitris drummondii
018 Callitris roei
116 Callitris roei
014 Callitris macleayana
080 Callitris macleayana
046 Callitris pancheri
037 Callitris pancheri
041 Callitris neocaledonica
036 Callitris neocaledonica
035 Callitris sulcata
040 Callitris sulcata
043 Fitzroya cupressoides
094 Fitzroya cupressoides
042 Diselma archeri
050 Widdringtonia schwarzii
049 Widdringtonia cedarbergensis
089 Austrocedrus chilensis
090 Austrocedrus chilensis
107 Libocedrus austrocaledonica
108 Libocedrus chevalieri
045 Libocedrus yateensis
106 Libocedrus bidwillii
093 Pilgerodendron uviferum
048 Pilgerodendron uviferum
047 Papuacedrus papuana
Callitroideae
060 Cupressus sempervirens
059 Cupressus funebris
097 Cupressus cashmeriana
102 Cupressus torulosa
100 Hesperocyparis macrocarpa
099 Hesperocyparis arizonica
063 Juniperus chinensis var sargentii
061 Juniperus chinensis
103 Juniperus deppeana
062 Juniperus rigida
104 Tetraclinis articulata
105 Tetraclinis articulata
069 Platycladus orientalis
096 Calocedrus decurrens
053 Calocedrus formosana
055 Chamaecyparis pisifera cv squarrosa
056 Chamaecyparis obtusa
071 Thuja occidentalis
067 Taxodium mucronatum
068 Taxodium distichum
057 Cryptomeria japonica
095 Sequoiadendron giganteum
065 Sequoia sempervirens
064 Metasequoia glyptostroboides
109 Athrotaxis selaginoides
066 Taiwania cryptomerioides
058 Cunninghamia lanceolata
0.02

Fig. S2 Maximum likelihood phylogeny of the nDNA dataset, estimated using an unpartitioned RAxML analysis. Bootstrap values ≥ 70 are shown at nodes. This tree is for Callitroideae only because sequences were not available for other Cupressaceae subfamilies.

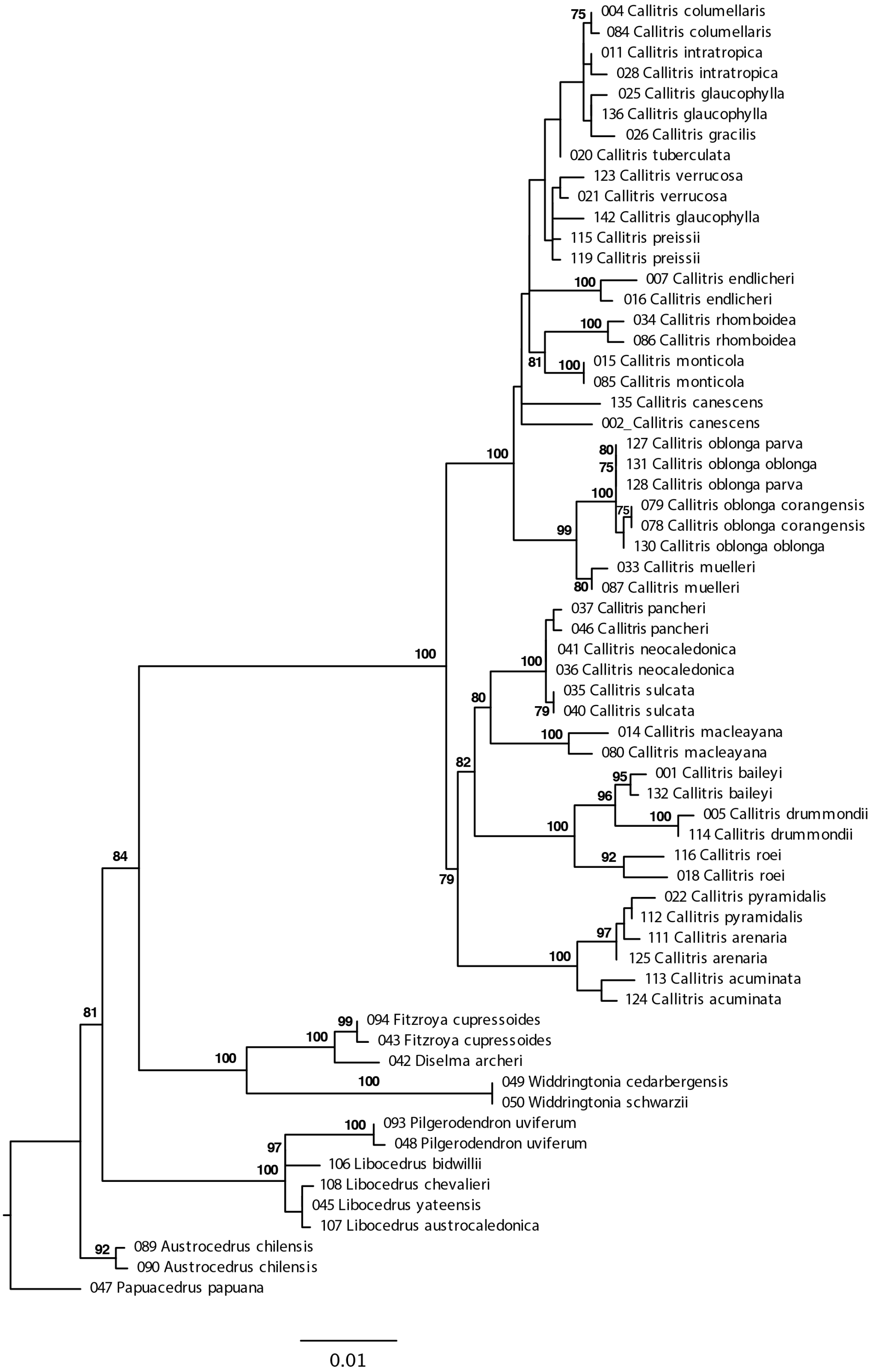
004 Callitris columellaris
084 Callitris columellaris
011 Callitris intratropica
028 Callitris intratropica
025 Callitris glaucophylla
136 Callitris glaucophylla
026 Callitris gracilis
020 Callitris tuberculata
123 Callitris verrucosa
021 Callitris verrucosa
142 Callitris glaucophylla
115 Callitris preissii
119 Callitris preissii
007 Callitris endlicheri
016 Callitris endlicheri
034 Callitris rhomboidea
086 Callitris rhomboidea
015 Callitris monticola
085 Callitris monticola
135 Callitris canescens
002_Callitris canescens
127 Callitris oblonga parva
131 Callitris oblonga oblonga
128 Callitris oblonga parva
079 Callitris oblonga corangensis
078 Callitris oblonga corangensis
130 Callitris oblonga oblonga
033 Callitris muelleri
087 Callitris muelleri
037 Callitris pancheri
046 Callitris pancheri
041 Callitris neocaledonica
036 Callitris neocaledonica
035 Callitris sulcata
040 Callitris sulcata
014 Callitris macleayana
080 Callitris macleayana
001 Callitris baileyi
132 Callitris baileyi
005 Callitris drummondii
114 Callitris drummondii
116 Callitris roei
018 Callitris roei
022 Callitris pyramidalis
112 Callitris pyramidalis
111 Callitris arenaria
125 Callitris arenaria
113 Callitris acuminata
124 Callitris acuminata
094 Fitzroya cupressoides
043 Fitzroya cupressoides
042 Diselma archeri
049 Widdringtonia cedarbergensis
050 Widdringtonia schwarzii
093 Pilgerodendron uviferum
048 Pilgerodendron uviferum
106 Libocedrus bidwillii
108 Libocedrus chevalieri
045 Libocedrus yateensis
107 Libocedrus austrocaledonica
089 Austrocedrus chilensis
090 Austrocedrus chilensis
047 Papuacedrus papuana
0.01

Fig. S3 Maximum likelihood phylogeny of combined cpDNA and nDNA datasets, using a partitioned RAxML analysis. Bootstrap values ≥ 70 are shown at nodes. Numbered circles at nodes indicate fossil-based calibrations listed in Table S3. Alternative placements, or omission, of nos 10 and 11 (shown in black on white backgrounds) were tested using Bayes Factors—for details, see text. The double-headed arrow indicates an alternative relationship between *Austrocedrus* and *Libocedrus* + *Pilgerodendron*. This was supported by the nDNA dataset (Fig. S2) and was used as a topological constraint in BEAST analyses (see Methods and Results in main text).

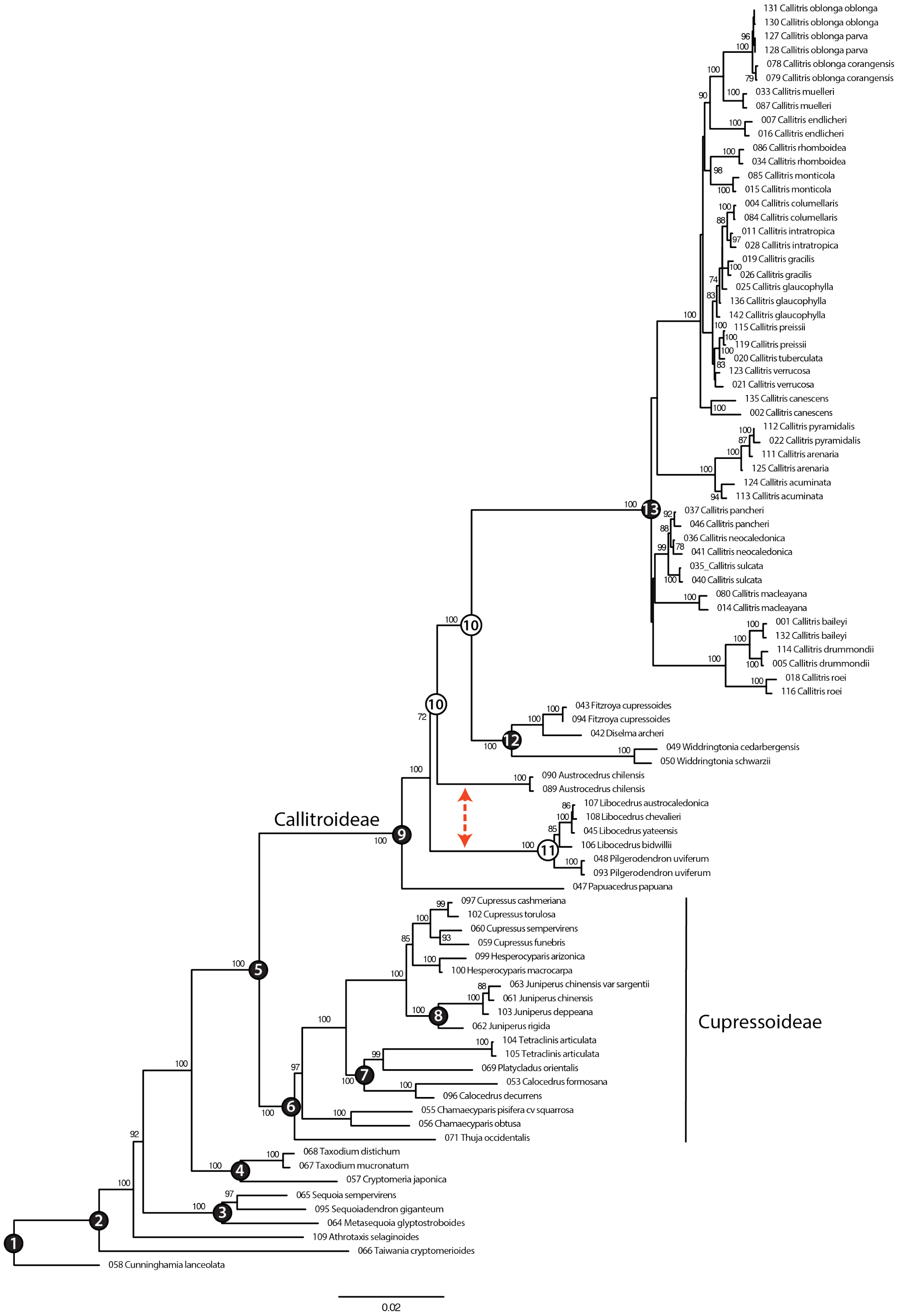
131 Callitris oblonga oblonga
130 Callitris oblonga oblonga
127 Callitris oblonga parva
128 Callitris oblonga parva
078 Callitris oblonga corangensis
079 Callitris oblonga corangensis
033 Callitris muelleri
087 Callitris muelleri
007 Callitris endlicheri
016 Callitris endlicheri
086 Callitris rhomboidea
034 Callitris rhomboidea
085 Callitris monticola
015 Callitris monticola
004 Callitris columellaris
084 Callitris columellaris
011 Callitris intratropica
028 Callitris intratropica
019 Callitris gracilis
026 Callitris gracilis
025 Callitris glaucophylla
136 Callitris glaucophylla
142 Callitris glaucophylla
115 Callitris preissii
119 Callitris preissii
020 Callitris tuberculata
123 Callitris verrucosa
021 Callitris verrucosa
135 Callitris canescens
002 Callitris canescens
112 Callitris pyramidalis
022 Callitris pyramidalis
111 Callitris arenaria
125 Callitris arenaria
124 Callitris acuminata
113 Callitris acuminata
037 Callitris pancheri
046 Callitris pancheri
036 Callitris neocaledonica
041 Callitris neocaledonica
035_Callitris sulcata
040 Callitris sulcata
080 Callitris macleayana
014 Callitris macleayana
001 Callitris baileyi
132 Callitris baileyi
114 Callitris drummondii
005 Callitris drummondii
018 Callitris roei
116 Callitris roei
043 Fitzroya cupressoides
094 Fitzroya cupressoides
042 Diselma archeri
049 Widdringtonia cedarbergensis
050 Widdringtonia schwarzii
090 Austrocedrus chilensis
089 Austrocedrus chilensis
107 Libocedrus austrocaledonica
108 Libocedrus chevalieri
045 Libocedrus yateensis
106 Libocedrus bidwillii
048 Pilgerodendron uviferum
093 Pilgerodendron uviferum
047 Papuacedrus papuana
Callitroideae
097 Cupressus cashmeriana
102 Cupressus torulosa
060 Cupressus sempervirens
059 Cupressus funebris
099 Hesperocyparis arizonica
100 Hesperocyparis macrocarpa
063 Juniperus chinensis var sargentii
061 Juniperus chinensis
103 Juniperus deppeana
062 Juniperus rigida
104 Tetraclinis articulata
105 Tetraclinis articulata
069 Platycladus orientalis
053 Calocedrus formosana
096 Calocedrus decurrens
055 Chamaecyparis pisifera cv squarrosa
056 Chamaecyparis obtusa
071 Thuja occidentalis
Cupressoideae
068 Taxodium distichum
067 Taxodium mucronatum
057 Cryptomeria japonica
065 Sequoia sempervirens
095 Sequoiadendron giganteum
064 Metasequoia glyptostroboides
109 Athrotaxis selaginoides
066 Taiwania cryptomerioides
058 Cunninghamia lanceolata
0.02

Fig. S4 Alternative placements of the c. 100-Myr-old *"Callitris octothamna"* fossil for calibrating the BEAST analyses of Cupressaceae. Phyllotaxis is reconstructed on the tree using parsimony; maximum likelihood mapping gave essentially the same result. The fossil has ternate phyllotaxis (as in most extant *Callitris* species), which could have originated at either node A (the *Callitris* stem node) or node B (the MRCA of *Austrocedrus* and *Callitris*). This is calibration 10 in Fig. S3 and Bayes Factors preferred its placement at node B. The tree is pruned to show only subfamilies Callitroideae and Cupressoideae. Only the states judged 'best' under the ML-Mk1 model are shown on branches and are mapped as polymorphic on branches where their likelihoods are not significantly different.

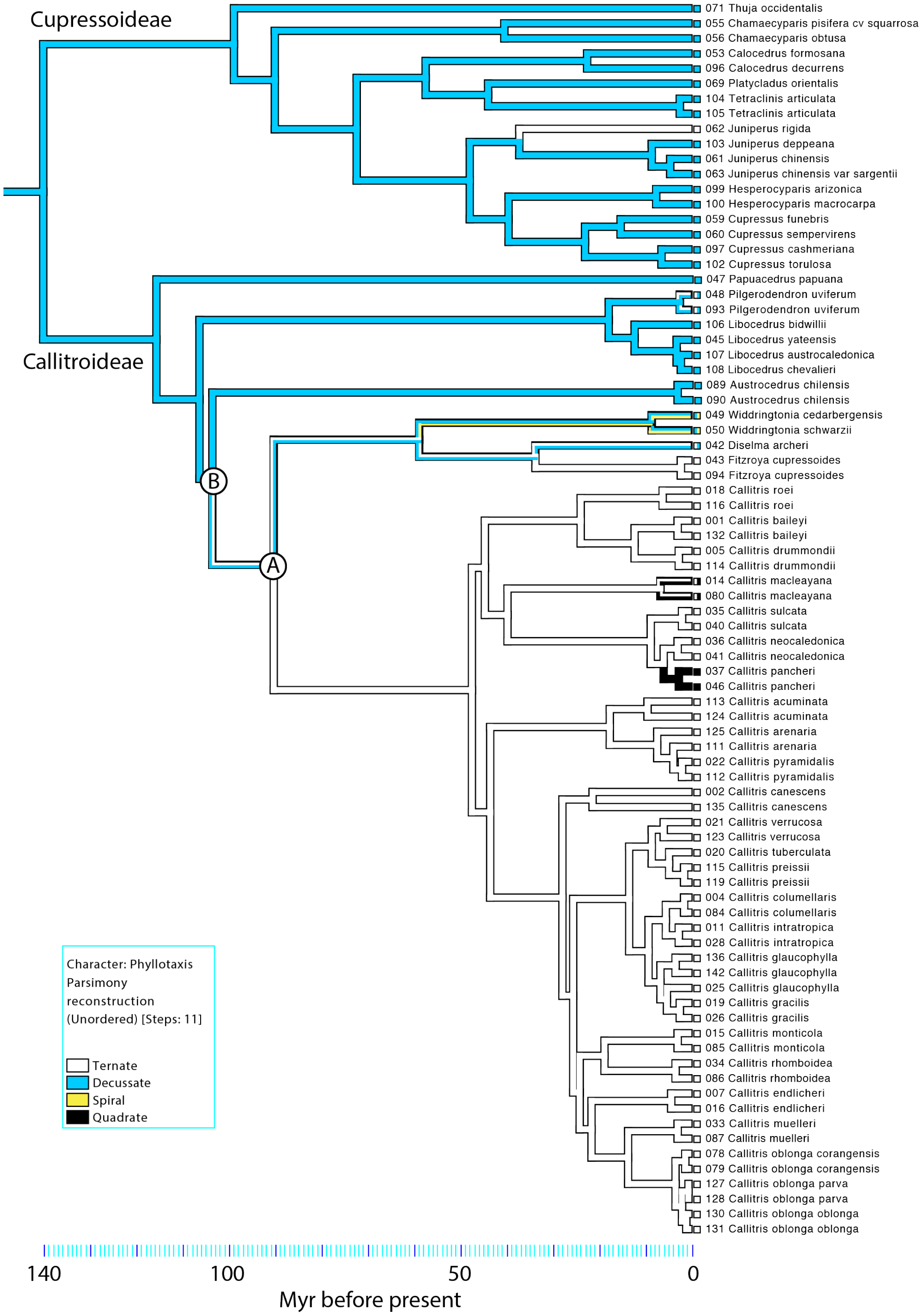

Cupressoideae
Callitroideae
071 Thuja occidentalis
055 Chamaecyparis pisifera cv squarrosa
056 Chamaecyparis obtusa
053 Calocedrus formosana
096 Calocedrus decurrens
069 Platycladus orientalis
104 Tetraclinis articulata
105 Tetraclinis articulata
062 Juniperus rigida
103 Juniperus deppeana
061 Juniperus chinensis
063 Juniperus chinensis var sargentii
099 Hesperocyparis arizonica
100 Hesperocyparis macrocarpa
059 Cupressus funebris
060 Cupressus sempervirens
097 Cupressus cashmeriana
102 Cupressus torulosa
047 Papuacedrus papuana
048 Pilgerodendron uviferum
093 Pilgerodendron uviferum
106 Libocedrus bidwillii
045 Libocedrus yateensis
107 Libocedrus austrocaledonica
108 Libocedrus chevalieri
089 Austrocedrus chilensis
090 Austrocedrus chilensis
049 Widdringtonia cedarbergensis
050 Widdringtonia schwarzii
042 Diselma archeri
043 Fitzroya cupressoides
094 Fitzroya cupressoides
018 Callitris roei
116 Callitris roei
001 Callitris baileyi
132 Callitris baileyi
005 Callitris drummondii
114 Callitris drummondii
014 Callitris macleayana
080 Callitris macleayana
035 Callitris sulcata
040 Callitris sulcata
036 Callitris neocaledonica
041 Callitris neocaledonica
037 Callitris pancheri
046 Callitris pancheri
113 Callitris acuminata
124 Callitris acuminata
125 Callitris arenaria
111 Callitris arenaria
022 Callitris pyramidalis
112 Callitris pyramidalis
002 Callitris canescens
135 Callitris canescens
021 Callitris verrucosa
123 Callitris verrucosa
020 Callitris tuberculata
115 Callitris preissii
119 Callitris preissii
004 Callitris columellaris
084 Callitris columellaris
011 Callitris intratropica
028 Callitris intratropica
136 Callitris glaucophylla
142 Callitris glaucophylla
025 Callitris glaucophylla
019 Callitris gracilis
026 Callitris gracilis
015 Callitris monticola
085 Callitris monticola
034 Callitris rhomboidea
086 Callitris rhomboidea
007 Callitris endlicheri
016 Callitris endlicheri
033 Callitris muelleri
087 Callitris muelleri
078 Callitris oblonga corangensis
079 Callitris oblonga corangensis
127 Callitris oblonga parva
128 Callitris oblonga parva
130 Callitris oblonga oblonga
131 Callitris oblonga oblonga
B
A
Character: Phyllotaxis
Parsimony
reconstruction
(Unordered) [Steps: 11]
Ternate
Decussate
Spiral
Quadrate
140
100
50
0
Myr before present

Fig. S5 Alternative placements of the c. 100-Myr-old *"Callitris octothamna"* fossil for calibrating BEAST analysis of Cupressaceae. Leaf dimorphism and monomorphism are reconstructed on the tree using parsimony; maximum likelihood mapping gave essentially the same result. The fossil has monomorphic leaves, a state which is shared with the *Widdringtonia-Callitris* clade and is homoplasious in *Pilgerodendron* and the *Cupressus-Juniperus* clade. This is calibration 10 in Fig. S3 and Bayes Factors preferred its placement at node B. The tree is pruned to show only subfamilies Callitroideae and Cupressoideae.

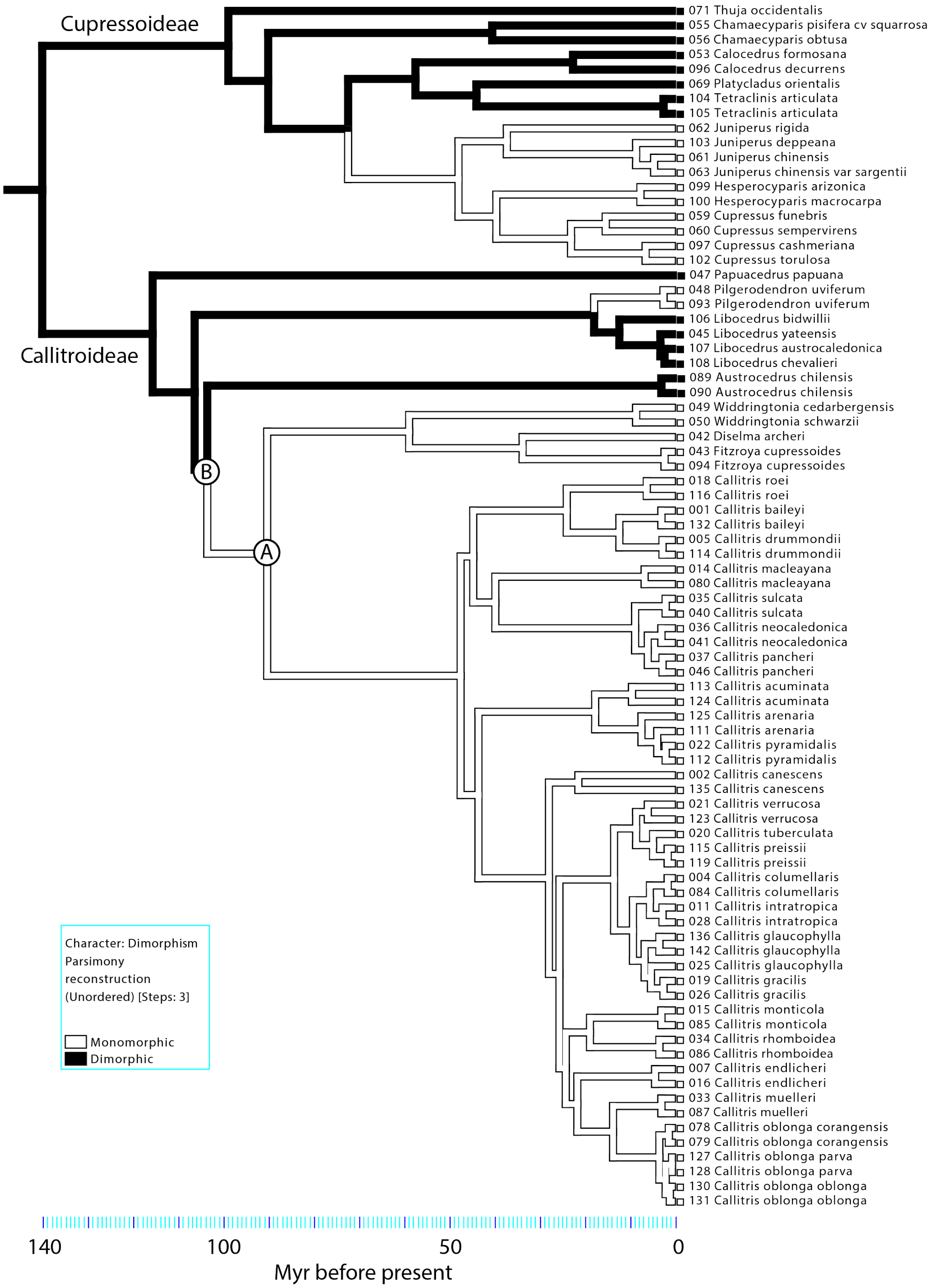
Cupressoideae
Callitroideae
071 Thuja occidentalis
055 Chamaecyparis pisifera cv squarrosa
056 Chamaecyparis obtusa
053 Calocedrus formosana
096 Calocedrus decurrens
069 Platycladus orientalis
104 Tetraclinis articulata
105 Tetraclinis articulata
062 Juniperus rigida
103 Juniperus deppeana
061 Juniperus chinensis
063 Juniperus chinensis var sargentii
099 Hesperocyparis arizonica
100 Hesperocyparis macrocarpa
059 Cupressus funebris
060 Cupressus sempervirens
097 Cupressus cashmeriana
102 Cupressus torulosa
047 Papuacedrus papuana
048 Pilgerodendron uviferum
093 Pilgerodendron uviferum
106 Libocedrus bidwillii
045 Libocedrus yateensis
107 Libocedrus austrocaledonica
108 Libocedrus chevalieri
089 Austrocedrus chilensis
090 Austrocedrus chilensis
049 Widdringtonia cedarbergensis
050 Widdringtonia schwarzii
042 Diselma archeri
043 Fitzroya cupressoides
094 Fitzroya cupressoides
018 Callitris roei
116 Callitris roei
001 Callitris baileyi
132 Callitris baileyi
005 Callitris drummondii
114 Callitris drummondii
014 Callitris macleayana
080 Callitris macleayana
035 Callitris sulcata
040 Callitris sulcata
036 Callitris neocaledonica
041 Callitris neocaledonica
037 Callitris pancheri
046 Callitris pancheri
113 Callitris acuminata
124 Callitris acuminata
125 Callitris arenaria
111 Callitris arenaria
022 Callitris pyramidalis
112 Callitris pyramidalis
002 Callitris canescens
135 Callitris canescens
021 Callitris verrucosa
123 Callitris verrucosa
020 Callitris tuberculata
115 Callitris preissii
119 Callitris preissii
004 Callitris columellaris
084 Callitris columellaris
011 Callitris intratropica
028 Callitris intratropica
136 Callitris glaucophylla
142 Callitris glaucophylla
025 Callitris glaucophylla
019 Callitris gracilis
026 Callitris gracilis
015 Callitris monticola
085 Callitris monticola
034 Callitris rhomboidea
086 Callitris rhomboidea
007 Callitris endlicheri
016 Callitris endlicheri
033 Callitris muelleri
087 Callitris muelleri
078 Callitris oblonga corangensis
079 Callitris oblonga corangensis
127 Callitris oblonga parva
128 Callitris oblonga parva
130 Callitris oblonga oblonga
131 Callitris oblonga oblonga
B
A
Character: Dimorphism
Parsimony
reconstruction
(Unordered) [Steps: 3]
Monomorphic
Dimorphic
140
100
50
0
Myr before present

Fig. S6 Alternative placements of the c. 100-Myr-old *"Callitris octothamna"* fossil for calibrating BEAST analysis of Cupressaceae. Cone-scale phyllotaxis is reconstructed on the tree using parsimony; maximum likelihood mapping gave essentially the same result. The fossil has two whorls of four scales, which is otherwise seen in paedomorphic extant *Callitris* species with quadrate leaves. Most *Callitris* species and some outgroups have two ternate whorls of cone scales. *"Callitris octothamna"* is unique in combining ternate leaves with quadrate cone scales; in all other known callitroids the cone-scales and leaf whorls have the same base number (2 or 3). Ternate cone scales could have originated at either node A (the *Callitris* stem node) or node B (the MRCA of *Austrocedrus* and *Callitris*). This is calibration 10 in Fig. S3 and Bayes Factors preferred its placement at node B. The tree is pruned to show only subfamilies Callitroideae and Cupressoideae. Only the states judged 'best' under the ML-Mk1 model are shown on branches and are mapped as polymorphic on branches where their likelihoods are not significantly different.

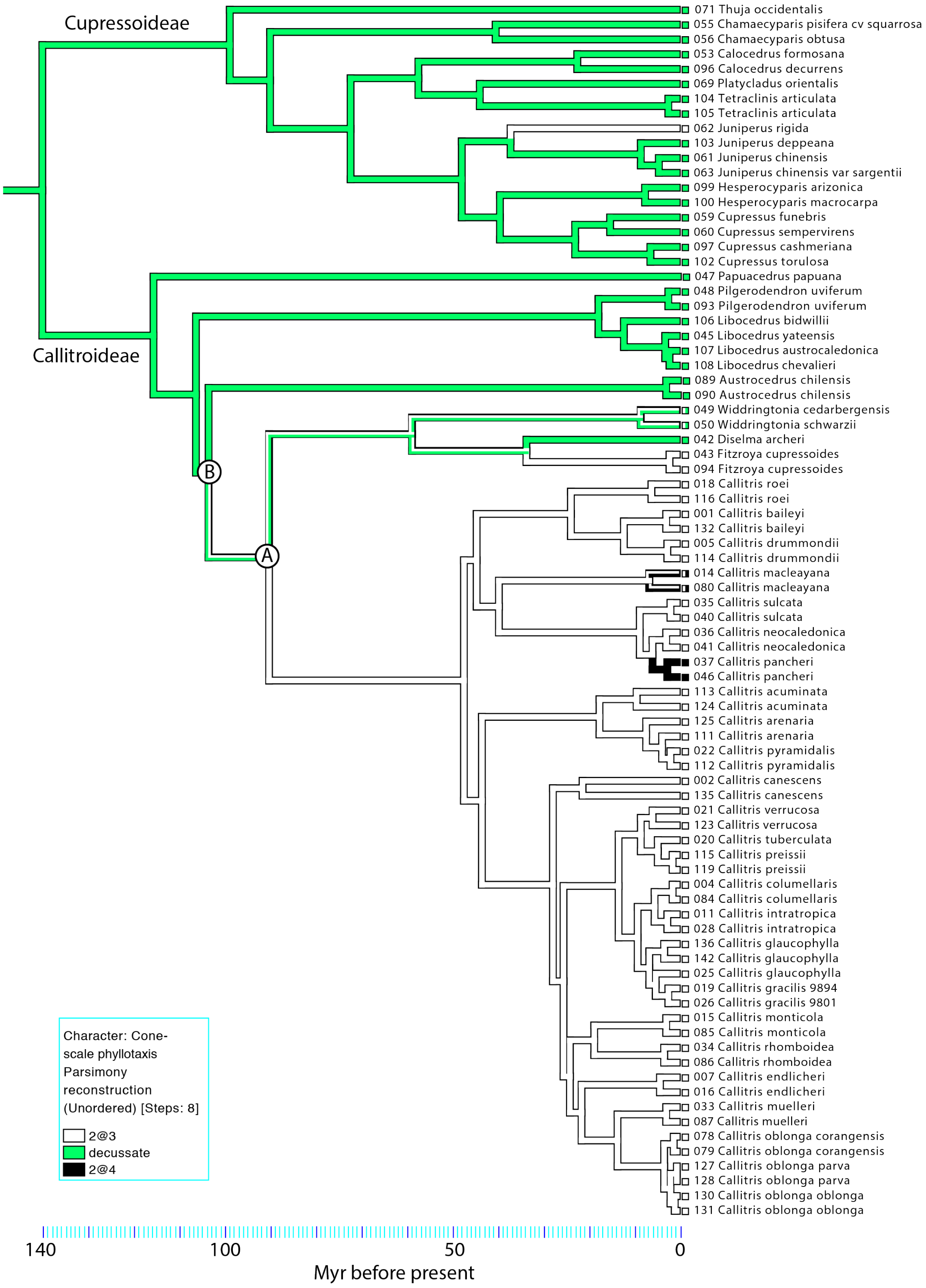

Cupressoideae
Callitroideae
071 Thuja occidentalis
055 Chamaecyparis pisifera cv squarrosa
056 Chamaecyparis obtusa
053 Calocedrus formosana
096 Calocedrus decurrens
069 Platycladus orientalis
104 Tetraclinis articulata
105 Tetraclinis articulata
062 Juniperus rigida
103 Juniperus deppeana
061 Juniperus chinensis
063 Juniperus chinensis var sargentii
099 Hesperocyparis arizonica
100 Hesperocyparis macrocarpa
059 Cupressus funebris
060 Cupressus sempervirens
097 Cupressus cashmeriana
102 Cupressus torulosa
047 Papuacedrus papuana
048 Pilgerodendron uviferum
093 Pilgerodendron uviferum
106 Libocedrus bidwillii
045 Libocedrus yateensis
107 Libocedrus austrocaledonica
108 Libocedrus chevalieri
089 Austrocedrus chilensis
090 Austrocedrus chilensis
049 Widdringtonia cedarbergensis
050 Widdringtonia schwarzii
042 Diselma archeri
043 Fitzroya cupressoides
094 Fitzroya cupressoides
018 Callitris roei
116 Callitris roei
001 Callitris baileyi
132 Callitris baileyi
005 Callitris drummondii
114 Callitris drummondii
014 Callitris macleayana
080 Callitris macleayana
035 Callitris sulcata
040 Callitris sulcata
036 Callitris neocaledonica
041 Callitris neocaledonica
037 Callitris pancheri
046 Callitris pancheri
113 Callitris acuminata
124 Callitris acuminata
125 Callitris arenaria
111 Callitris arenaria
022 Callitris pyramidalis
112 Callitris pyramidalis
002 Callitris canescens
135 Callitris canescens
021 Callitris verrucosa
123 Callitris verrucosa
020 Callitris tuberculata
115 Callitris preissii
119 Callitris preissii
004 Callitris columellaris
084 Callitris columellaris
011 Callitris intratropica
028 Callitris intratropica
136 Callitris glaucophylla
142 Callitris glaucophylla
025 Callitris glaucophylla
019 Callitris gracilis 9894
026 Callitris gracilis 9801
015 Callitris monticola
085 Callitris monticola
034 Callitris rhomboidea
086 Callitris rhomboidea
007 Callitris endlicheri
016 Callitris endlicheri
033 Callitris muelleri
087 Callitris muelleri
078 Callitris oblonga corangensis
079 Callitris oblonga corangensis
127 Callitris oblonga parva
128 Callitris oblonga parva
130 Callitris oblonga oblonga
131 Callitris oblonga oblonga
B
A
Character: Cone-scale phyllotaxis
Parsimony reconstruction
(Unordered) [Steps: 8]
2@3
decussate
2@4
140
100
50
0
Myr before present

Fig. S7 Time tree of Cupressaceae estimated from the combined cpDNA-nDNA dataset, using a partitioned BEAST analysis and calibrated using lognormal priors with all fossils shown in Fig. S3 except no. 11 (*Libocedrus mesibovii*). Posterior probabilities ≥ 70 are shown at nodes. Node bars indicate 95% HPD intervals of posterior diverence times. Node 10 was calibrated with the *"Callitris octothamna"* fossil but not given a monophyly constraint (contrast Fig. S10).

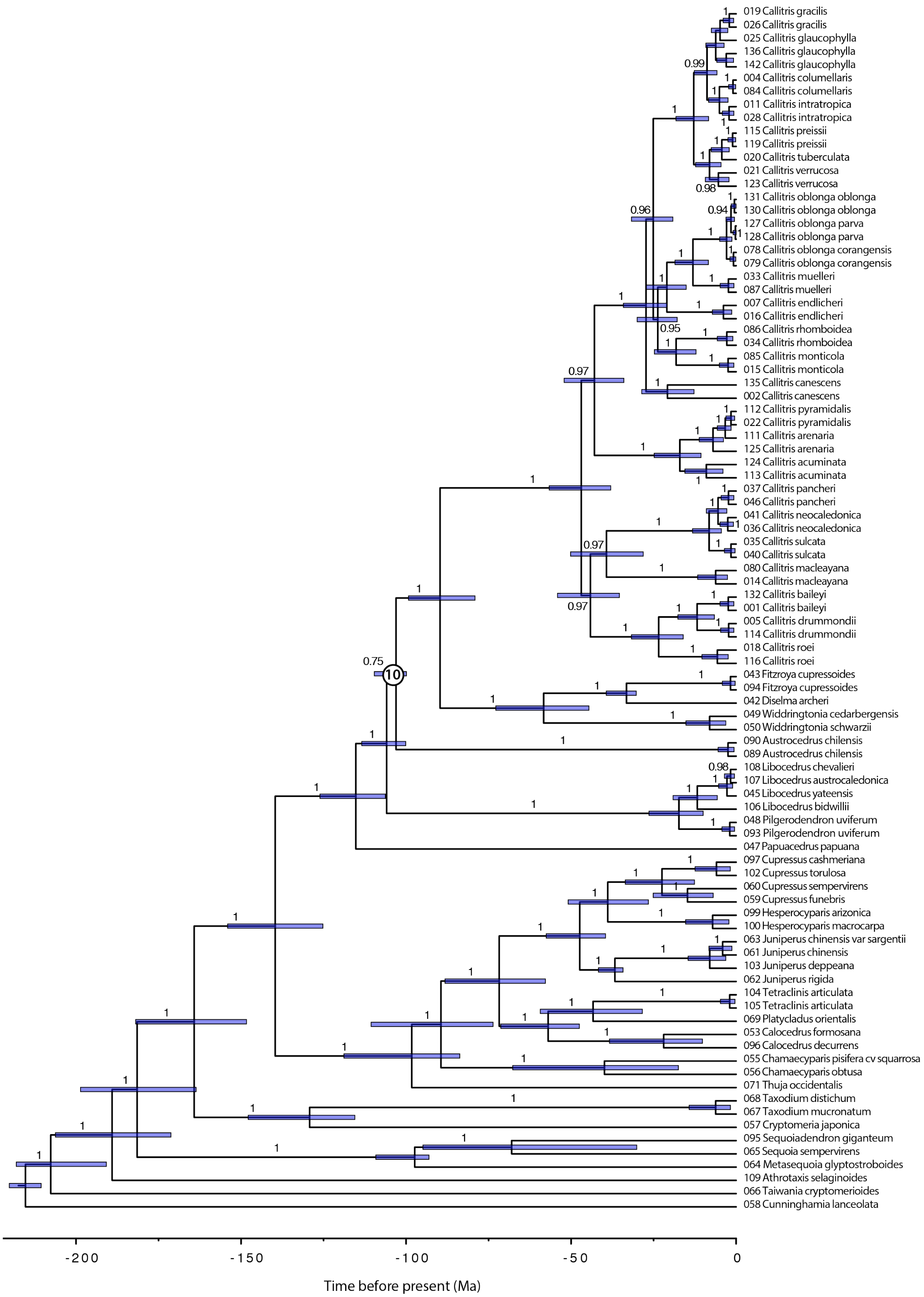
019 Callitris gracilis
026 Callitris gracilis
025 Callitris glaucophylla
136 Callitris glaucophylla
142 Callitris glaucophylla
004 Callitris columellaris
084 Callitris columellaris
011 Callitris intratropica
028 Callitris intratropica
115 Callitris preissii
119 Callitris preissii
020 Callitris tuberculata
021 Callitris verrucosa
123 Callitris verrucosa
131 Callitris oblonga oblonga
130 Callitris oblonga oblonga
127 Callitris oblonga parva
128 Callitris oblonga parva
078 Callitris oblonga corangensis
079 Callitris oblonga corangensis
033 Callitris muelleri
087 Callitris muelleri
007 Callitris endlicheri
016 Callitris endlicheri
086 Callitris rhomboidea
034 Callitris rhomboidea
085 Callitris monticola
015 Callitris monticola
135 Callitris canescens
002 Callitris canescens
112 Callitris pyramidalis
022 Callitris pyramidalis
111 Callitris arenaria
125 Callitris arenaria
124 Callitris acuminata
113 Callitris acuminata
037 Callitris pancheri
046 Callitris pancheri
041 Callitris neocaledonica
036 Callitris neocaledonica
035 Callitris sulcata
040 Callitris sulcata
080 Callitris macleayana
014 Callitris macleayana
132 Callitris baileyi
001 Callitris baileyi
005 Callitris drummondii
114 Callitris drummondii
018 Callitris roei
116 Callitris roei
043 Fitzroya cupressoides
094 Fitzroya cupressoides
042 Diselma archeri
049 Widdringtonia cedarbergensis
050 Widdringtonia schwarzii
090 Austrocedrus chilensis
089 Austrocedrus chilensis
108 Libocedrus chevalieri
107 Libocedrus austrocaledonica
045 Libocedrus yateensis
106 Libocedrus bidwillii
048 Pilgerodendron uviferum
093 Pilgerodendron uviferum
047 Papuacedrus papuana
097 Cupressus cashmeriana
102 Cupressus torulosa
060 Cupressus sempervirens
059 Cupressus funebris
099 Hesperocyparis arizonica
100 Hesperocyparis macrocarpa
063 Juniperus chinensis var sargentii
061 Juniperus chinensis
103 Juniperus deppeana
062 Juniperus rigida
104 Tetraclinis articulata
105 Tetraclinis articulata
069 Platycladus orientalis
053 Calocedrus formosana
096 Calocedrus decurrens
055 Chamaecyparis pisifera cv squarrosa
056 Chamaecyparis obtusa
071 Thuja occidentalis
068 Taxodium distichum
067 Taxodium mucronatum
057 Cryptomeria japonica
095 Sequoiadendron giganteum
065 Sequoia sempervirens
064 Metasequoia glyptostroboides
109 Athrotaxis selaginoides
066 Taiwania cryptomerioides
058 Cunninghamia lanceolata
0.99
0.98
0.96
0.94
0.95
0.97
0.97
0.97
0.75
10
0.98
-200
-150
-100
-50
0
Time before present (Ma)

Fig. S8 Parsimony reconstruction of the trait cone-serotiny. This tree was generated in BEAST with calibrations including *"Callitris octothamna"* at the MRCA of *Austrocedrus* and *Callitris*. The Tasmanian fossil *C. leaensis* has been inserted by hand mid-way along the stem-lineage of *Callitris*, with its branch terminating at c. 30 Ma, which is the age of the fossil. Grey shading indicates branches with ambiguous trait reconstruction. ML mapping gave essentially the same result.

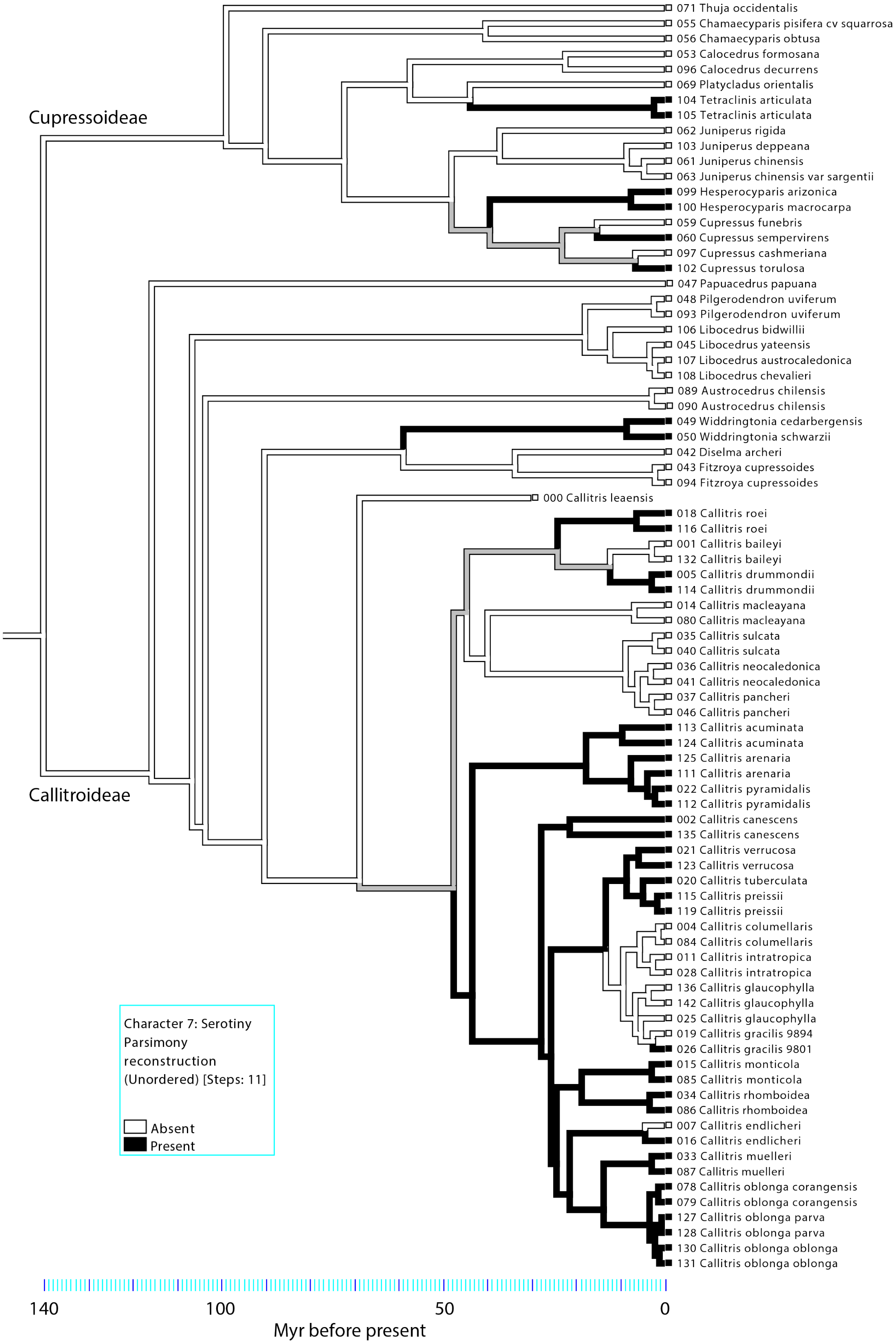

071 Thuja occidentalis
055 Chamaecyparis pisifera cv squarrosa
056 Chamaecyparis obtusa
053 Calocedrus formosana
096 Calocedrus decurrens
069 Platycladus orientalis
104 Tetraclinis articulata
105 Tetraclinis articulata
062 Juniperus rigida
103 Juniperus deppeana
061 Juniperus chinensis
063 Juniperus chinensis var sargentii
099 Hesperocyparis arizonica
100 Hesperocyparis macrocarpa
059 Cupressus funebris
060 Cupressus sempervirens
097 Cupressus cashmeriana
102 Cupressus torulosa
047 Papuacedrus papuana
048 Pilgerodendron uviferum
093 Pilgerodendron uviferum
106 Libocedrus bidwillii
045 Libocedrus yateensis
107 Libocedrus austrocaledonica
108 Libocedrus chevalieri
089 Austrocedrus chilensis
090 Austrocedrus chilensis
049 Widdringtonia cedarbergensis
050 Widdringtonia schwarzii
042 Diselma archeri
043 Fitzroya cupressoides
094 Fitzroya cupressoides
000 Callitris leaensis
018 Callitris roei
116 Callitris roei
001 Callitris baileyi
132 Callitris baileyi
005 Callitris drummondii
114 Callitris drummondii
014 Callitris macleayana
080 Callitris macleayana
035 Callitris sulcata
040 Callitris sulcata
036 Callitris neocaledonica
041 Callitris neocaledonica
037 Callitris pancheri
046 Callitris pancheri
113 Callitris acuminata
124 Callitris acuminata
125 Callitris arenaria
111 Callitris arenaria
022 Callitris pyramidalis
112 Callitris pyramidalis
002 Callitris canescens
135 Callitris canescens
021 Callitris verrucosa
123 Callitris verrucosa
020 Callitris tuberculata
115 Callitris preissii
119 Callitris preissii
004 Callitris columellaris
084 Callitris columellaris
011 Callitris intratropica
028 Callitris intratropica
136 Callitris glaucophylla
142 Callitris glaucophylla
025 Callitris glaucophylla
019 Callitris gracilis 9894
026 Callitris gracilis 9801
015 Callitris monticola
085 Callitris monticola
034 Callitris rhomboidea
086 Callitris rhomboidea
007 Callitris endlicheri
016 Callitris endlicheri
033 Callitris muelleri
087 Callitris muelleri
078 Callitris oblonga corangensis
079 Callitris oblonga corangensis
127 Callitris oblonga parva
128 Callitris oblonga parva
130 Callitris oblonga oblonga
131 Callitris oblonga oblonga
Cupressoideae
Callitroideae
Character 7: Serotiny
Parsimony
reconstruction
(Unordered) [Steps: 11]
Absent
Present
140
100
50
0
Myr before present

Fig. S9 Parsimony reconstruction of the trait habitat fire-prone. This tree was generated in BEAST with calibrations including *"Callitris octothamna"* at the MRCA of *Austrocedrus* and *Callitris*. The Tasmanian fossil *C. leaensis* has been inserted by hand mid-way along the stem-lineage of *Callitris*, with its branch terminating at c. 30 Ma, which is the age of the fossil. ML mapping gave essentially the same result.

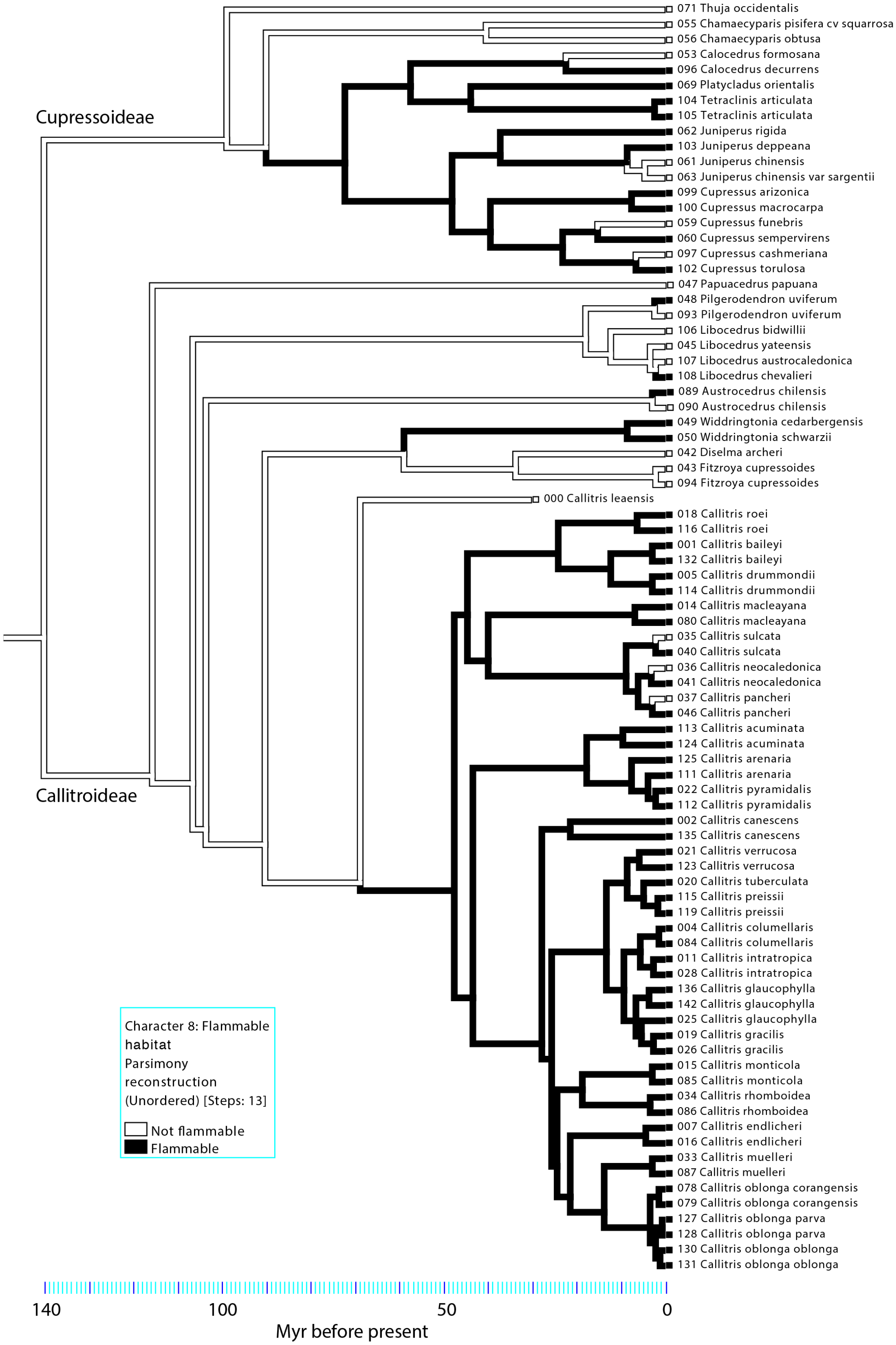

Cupressoideae
Callitroideae
071 Thuja occidentalis
055 Chamaecyparis pisifera cv squarrosa
056 Chamaecyparis obtusa
053 Calocedrus formosana
096 Calocedrus decurrens
069 Platycladus orientalis
104 Tetraclinis articulata
105 Tetraclinis articulata
062 Juniperus rigida
103 Juniperus deppeana
061 Juniperus chinensis
063 Juniperus chinensis var sargentii
099 Cupressus arizonica
100 Cupressus macrocarpa
059 Cupressus funebris
060 Cupressus sempervirens
097 Cupressus cashmeriana
102 Cupressus torulosa
047 Papuacedrus papuana
048 Pilgerodendron uviferum
093 Pilgerodendron uviferum
106 Libocedrus bidwillii
045 Libocedrus yateensis
107 Libocedrus austrocaledonica
108 Libocedrus chevalieri
089 Austrocedrus chilensis
090 Austrocedrus chilensis
049 Widdringtonia cedarbergensis
050 Widdringtonia schwarzii
042 Diselma archeri
043 Fitzroya cupressoides
094 Fitzroya cupressoides
000 Callitris leaensis
018 Callitris roei
116 Callitris roei
001 Callitris baileyi
132 Callitris baileyi
005 Callitris drummondii
114 Callitris drummondii
014 Callitris macleayana
080 Callitris macleayana
035 Callitris sulcata
040 Callitris sulcata
036 Callitris neocaledonica
041 Callitris neocaledonica
037 Callitris pancheri
046 Callitris pancheri
113 Callitris acuminata
124 Callitris acuminata
125 Callitris arenaria
111 Callitris arenaria
022 Callitris pyramidalis
112 Callitris pyramidalis
002 Callitris canescens
135 Callitris canescens
021 Callitris verrucosa
123 Callitris verrucosa
020 Callitris tuberculata
115 Callitris preissii
119 Callitris preissii
004 Callitris columellaris
084 Callitris columellaris
011 Callitris intratropica
028 Callitris intratropica
136 Callitris glaucophylla
142 Callitris glaucophylla
025 Callitris glaucophylla
019 Callitris gracilis
026 Callitris gracilis
015 Callitris monticola
085 Callitris monticola
034 Callitris rhomboidea
086 Callitris rhomboidea
007 Callitris endlicheri
016 Callitris endlicheri
033 Callitris muelleri
087 Callitris muelleri
078 Callitris oblonga corangensis
079 Callitris oblonga corangensis
127 Callitris oblonga parva
128 Callitris oblonga parva
130 Callitris oblonga oblonga
131 Callitris oblonga oblonga
Character 8: Flammable habitat
Parsimony reconstruction
(Unordered) [Steps: 13]
Not flammable
Flammable
140
100
50
0
Myr before present

Fig. S10 Time tree of Cupressaceae estimated from the combined cpDNA-nDNA dataset, using a partitioned BEAST analysis and calibrated using lognormal priors with all fossils shown in Fig. S3 except *Libocedrus* (11). Posterior probabilities ≥ 70 are shown at nodes. Node bars indicate 95% HPD intervals of posterior divergence times. The node numbered 10 (MRCA of *Pilgerodendron* and *Callitris*) was given a monophyly constraint in BEAST and calibrated with the *"Callitris octothamna"* fossil (contrast Fig. S7).

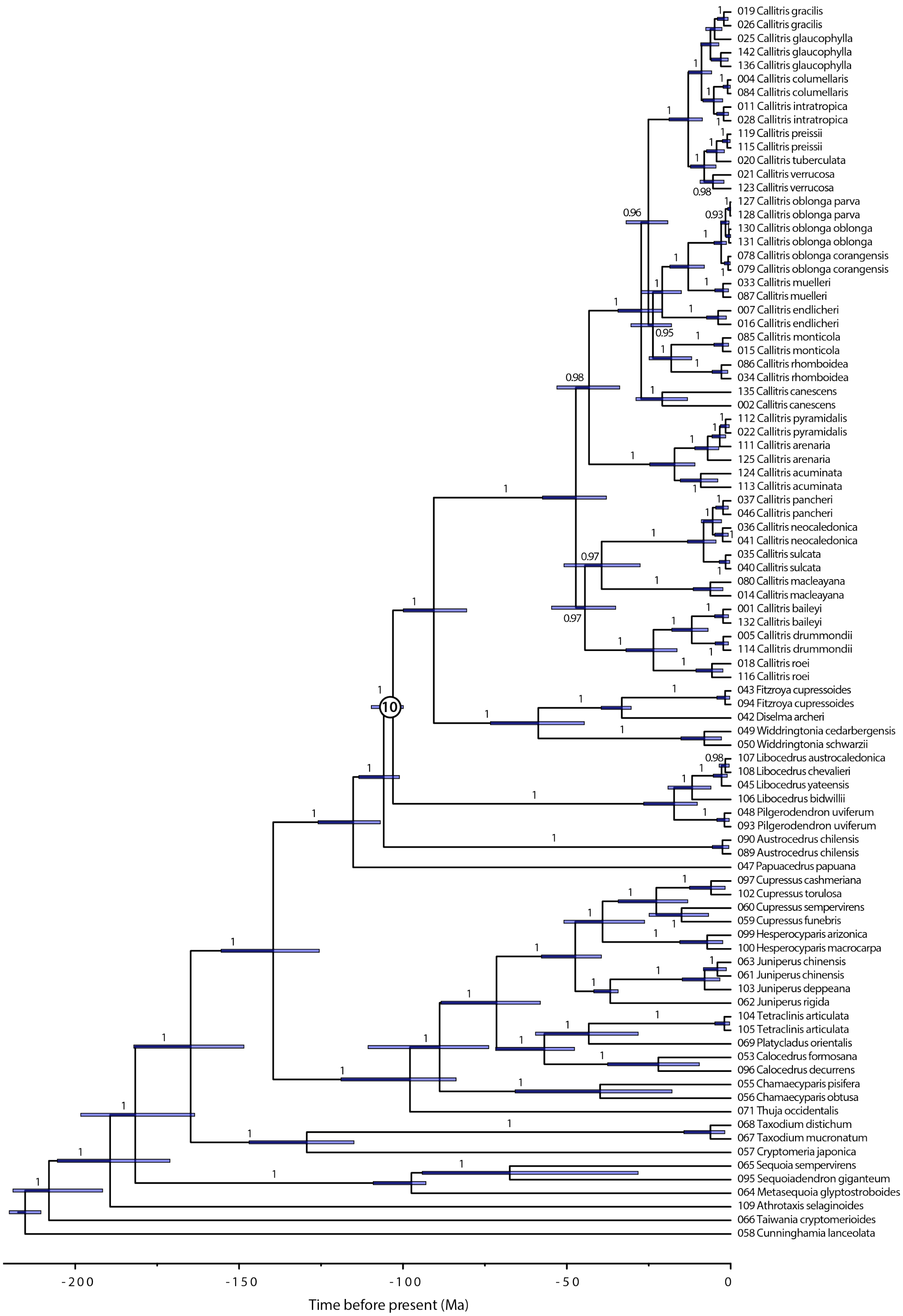

019 Callitris gracilis
026 Callitris gracilis
025 Callitris glaucophylla
142 Callitris glaucophylla
136 Callitris glaucophylla
004 Callitris columellaris
084 Callitris columellaris
011 Callitris intratropica
028 Callitris intratropica
119 Callitris preissii
115 Callitris preissii
020 Callitris tuberculata
021 Callitris verrucosa
123 Callitris verrucosa
127 Callitris oblonga parva
128 Callitris oblonga parva
130 Callitris oblonga oblonga
131 Callitris oblonga oblonga
078 Callitris oblonga corangensis
079 Callitris oblonga corangensis
033 Callitris muelleri
087 Callitris muelleri
007 Callitris endlicheri
016 Callitris endlicheri
085 Callitris monticola
015 Callitris monticola
086 Callitris rhomboidea
034 Callitris rhomboidea
135 Callitris canescens
002 Callitris canescens
112 Callitris pyramidalis
022 Callitris pyramidalis
111 Callitris arenaria
125 Callitris arenaria
124 Callitris acuminata
113 Callitris acuminata
037 Callitris pancheri
046 Callitris pancheri
036 Callitris neocaledonica
041 Callitris neocaledonica
035 Callitris sulcata
040 Callitris sulcata
080 Callitris macleayana
014 Callitris macleayana
001 Callitris baileyi
132 Callitris baileyi
005 Callitris drummondii
114 Callitris drummondii
018 Callitris roei
116 Callitris roei
043 Fitzroya cupressoides
094 Fitzroya cupressoides
042 Diselma archeri
049 Widdringtonia cedarbergensis
050 Widdringtonia schwarzii
107 Libocedrus austrocaledonica
108 Libocedrus chevalieri
045 Libocedrus yateensis
106 Libocedrus bidwillii
048 Pilgerodendron uviferum
093 Pilgerodendron uviferum
090 Austrocedrus chilensis
089 Austrocedrus chilensis
047 Papuacedrus papuana
097 Cupressus cashmeriana
102 Cupressus torulosa
060 Cupressus sempervirens
059 Cupressus funebris
099 Hesperocyparis arizonica
100 Hesperocyparis macrocarpa
063 Juniperus chinensis
061 Juniperus chinensis
103 Juniperus deppeana
062 Juniperus rigida
104 Tetraclinis articulata
105 Tetraclinis articulata
069 Platycladus orientalis
053 Calocedrus formosana
096 Calocedrus decurrens
055 Chamaecyparis pisifera
056 Chamaecyparis obtusa
071 Thuja occidentalis
068 Taxodium distichum
067 Taxodium mucronatum
057 Cryptomeria japonica
065 Sequoia sempervirens
095 Sequoiadendron giganteum
064 Metasequoia glyptostroboides
109 Athrotaxis selaginoides
066 Taiwania cryptomerioides
058 Cunninghamia lanceolata
10
-200
-150
-100
-50
0
Time before present (Ma)

Fig. S11 Time tree of Cupressaceae estimated from the combined cpDNA-nDNA dataset, using a partitioned BEAST analysis and calibrated (using lognormal priors) with all fossils shown in Fig. S3 except no. 11 (*Libocedrus mesibovii*) and *"Callitris octothamna"* (10). Posterior probabilities ≥ 70 are shown at nodes. Node bars indicate 95% HPD intervals of posterior diverence times. Node 10 was not given a monophyly constraint; note the relatively weak posterior probability (0.83).

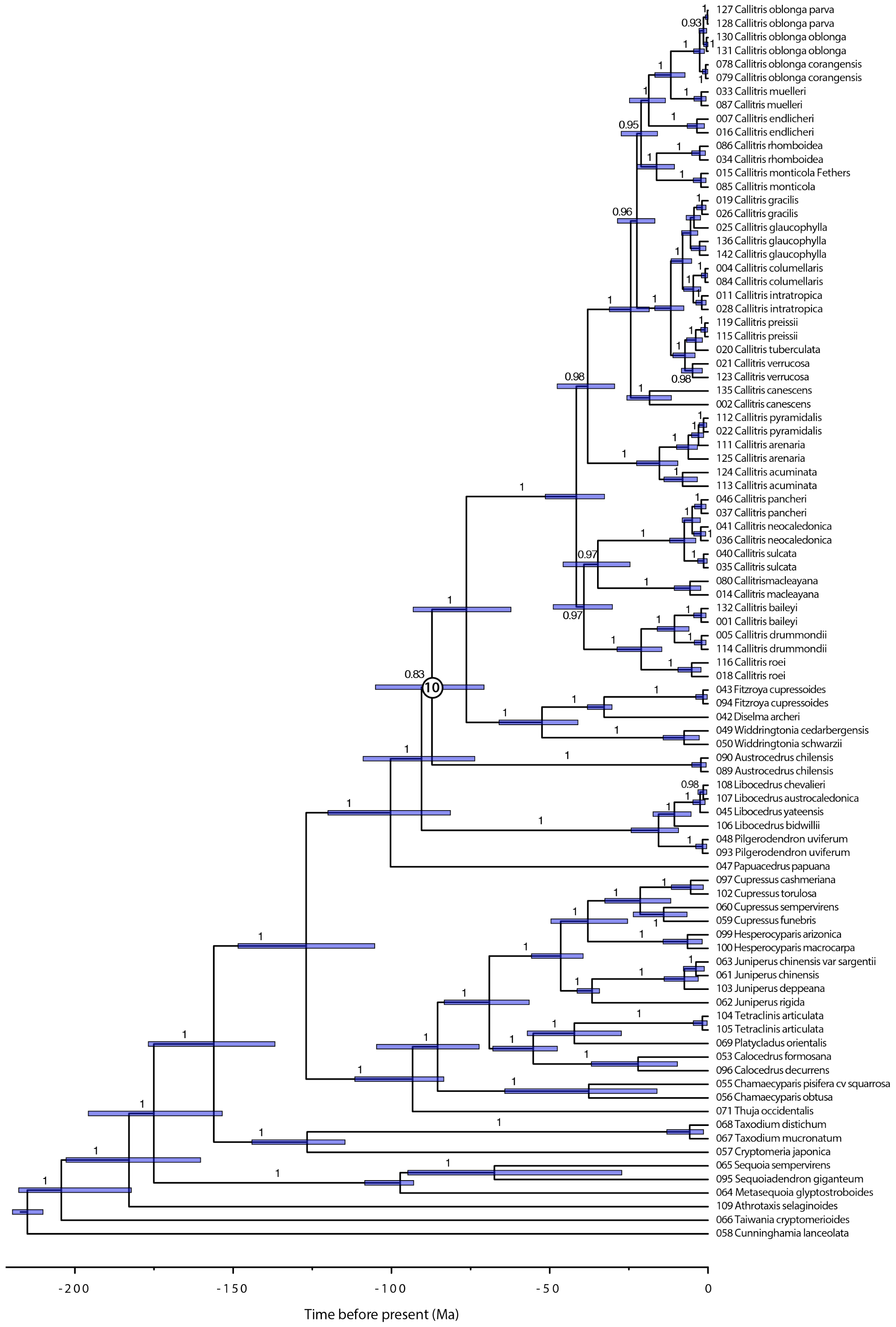
127 Callitris oblonga parva
128 Callitris oblonga parva
130 Callitris oblonga oblonga
131 Callitris oblonga oblonga
078 Callitris oblonga corangensis
079 Callitris oblonga corangensis
033 Callitris muelleri
087 Callitris muelleri
007 Callitris endlicheri
016 Callitris endlicheri
086 Callitris rhomboidea
034 Callitris rhomboidea
015 Callitris monticola Fethers
085 Callitris monticola
019 Callitris gracilis
026 Callitris gracilis
025 Callitris glaucophylla
136 Callitris glaucophylla
142 Callitris glaucophylla
004 Callitris columellaris
084 Callitris columellaris
011 Callitris intratropica
028 Callitris intratropica
119 Callitris preissii
115 Callitris preissii
020 Callitris tuberculata
021 Callitris verrucosa
123 Callitris verrucosa
135 Callitris canescens
002 Callitris canescens
112 Callitris pyramidalis
022 Callitris pyramidalis
111 Callitris arenaria
125 Callitris arenaria
124 Callitris acuminata
113 Callitris acuminata
046 Callitris pancheri
037 Callitris pancheri
041 Callitris neocaledonica
036 Callitris neocaledonica
040 Callitris sulcata
035 Callitris sulcata
080 Callitrismacleayana
014 Callitris macleayana
132 Callitris baileyi
001 Callitris baileyi
005 Callitris drummondii
114 Callitris drummondii
116 Callitris roei
018 Callitris roei
043 Fitzroya cupressoides
094 Fitzroya cupressoides
042 Diselma archeri
049 Widdringtonia cedarbergensis
050 Widdringtonia schwarzii
090 Austrocedrus chilensis
089 Austrocedrus chilensis
108 Libocedrus chevalieri
107 Libocedrus austrocaledonica
045 Libocedrus yateensis
106 Libocedrus bidwillii
048 Pilgerodendron uviferum
093 Pilgerodendron uviferum
047 Papuacedrus papuana
097 Cupressus cashmeriana
102 Cupressus torulosa
060 Cupressus sempervirens
059 Cupressus funebris
099 Hesperocyparis arizonica
100 Hesperocyparis macrocarpa
063 Juniperus chinensis var sargentii
061 Juniperus chinensis
103 Juniperus deppeana
062 Juniperus rigida
104 Tetraclinis articulata
105 Tetraclinis articulata
069 Platycladus orientalis
053 Calocedrus formosana
096 Calocedrus decurrens
055 Chamaecyparis pisifera cv squarrosa
056 Chamaecyparis obtusa
071 Thuja occidentalis
068 Taxodium distichum
067 Taxodium mucronatum
057 Cryptomeria japonica
065 Sequoia sempervirens
095 Sequoiadendron giganteum
064 Metasequoia glyptostroboides
109 Athrotaxis selaginoides
066 Taiwania cryptomerioides
058 Cunninghamia lanceolata
10
-200
-150
-100
-50
0
Time before present (Ma)

Table S1 Taxa sampled, sample sources, their geographic origin and GenBank accession numbers of all sequences included in this study. All sequences were newly generated for this study. Taxonomy follows Hill (1998), Piggin & Bruhl (2010) and Byng (2015) for Callitris s.l. and Farjon (2005) and de Laubenfels et al. (2012) for the rest of Cupressaceae. Herbarium acronyms follow Index Herbariorum at http://sciweb.nybg.org/science2/. Crisp vouchers are all in CANB. Sources of some samples are detailed in Sakaguchi et al. (2013), as cited. Abbreviations of Australian states: NSW, New South Wales; NT, Northern Territory; Qld, Queensland; SA, South Australia; Tas, Tasmania; Vic, Victoria; WA, Western Australia.

Part 1: Chloroplast DNA sequences

Taxon	Sample number, voucher (Herbarium) or source	*Geographic origin*	*rbcL* gene	*matK* gene	matK–trnK	rpl16	psbH–psbB
**Cupressaceae subfam. Callitroideae**							
Austrocedrus chilensis (D.Don) Florin & Boutelje	089, Crisp 10667	Argentina	LC405318	LC404776	LC404915	LC405457	LC405188
	090, Crisp 10669	Argentina	LC405319	LC404777	LC404916	LC405458	LC405189
Callitris acuminata (Parl.) F.Muell.	113, Crisp 10908	WA	LC405340	LC404798	LC404938	LC405481	LC405212
	124, Crisp 11186	WA	LC405351	LC404809	LC404949	LC405492	LC405223
C. arenaria (C.A.Gardner) J.E.Piggin & J.J. Bruhl	111, Crisp 10803	WA	LC405338	LC404796	LC404936	LC405479	LC405210
	125, Crisp 11187	WA	LC405352	LC404810	LC404950	LC405493	LC405224
C. baileyi C.T.White	132, Crisp 11289A	Qld	LC405359	LC404817	LC404957	LC405500	LC405231
	001, Crisp 10153A	Qld	LC405243	LC404698	LC404829	LC405371	LC405110
C. canescens (Parl.) S.T.Blake	002, Crisp 10118	SA	LC405244	LC404699	LC404830	LC405372	LC405111
	135, Crisp 9703	WA	LC405362	LC404820	LC404960	LC405503	LC405234
C. columellaris F.Muell.	004, Crisp 10321	Qld	LC405246	LC404701	LC404832	LC405374	LC405113
	084, Crisp 10703	NSW	LC405313	LC404771	LC404910	LC405452	LC405183
C. drummondii (Parl.) Benth. & Hook.f. ex F.Muell.	114, Crisp 10998	WA	LC405341	LC404799	LC404939	LC405482	LC405213
	005, Crisp 9945	WA	LC405247	LC404702	LC404833	LC405375	LC405114

C. endlicheri (Parl.) F.M.Bailey	007, Crisp 10345	Qld	LC405249	LC404704	LC404835	LC405377	LC405116
	016, Crisp 10340	Qld	LC405258	LC404713	LC404844	LC405386	LC405125
C. glaucophylla Joy Thomps. & L.A.S.Johnson	025, Crisp 9796	NSW	LC405265	LC404720	LC404851	LC405393	LC405132
	136, Central Australia, CA1-1 (Sakaguchi *et al.*, 2013)	NT	LC405363	LC404821	LC404961	LC405504	LC405235
	142, WA3-1 (Sakaguchi *et al.*, 2013)	WA	LC405369	LC404827	LC404967	LC405510	LC405241
C. gracilis R.T.Baker	026, Crisp 9801	SA	LC405266	LC404721	LC404852	LC405394	LC405133
	019, Crisp 9894	WA	LC405260	LC404715	LC404846	LC405388	LC405127
C. intratropica R.T.Baker & H.G.Sm.	028, Crisp 9760	NT	LC405268	LC404723	LC404854	LC405396	LC405135
	011, Crisp 10221	Qld	LC405253	LC404708	LC404839	LC405381	LC405120
C. macleayana (F.Muell.) F.Muell.	014, Crisp 10138	NSW	LC405256	LC404711	LC404842	LC405384	LC405123
	080, Mt Windsor 2 (Sakaguchi *et al.*, 2013)	Qld	LC405309	LC404767	LC404906	LC405448	LC405179
C. monticola J.Garden	085, Crisp 10707	NSW	LC405314	LC404772	LC404911	LC405453	LC405184
	015, Fethers 42 (CANB)		LC405257	LC404712	LC404843	LC405385	LC405124
C. muelleri (Parl.) Benth. & Hook.f. ex F.Muell.	033, Beesley 281 (CANB)	NSW	LC405273	LC404728	LC404859	LC405401	LC405140
	087, Blue Mts B (Sakaguchi *et al.*, 2013)	NSW	LC405316	LC404774	LC404913	LC405455	LC405186
C. neocaledonica Dümmer	036, cult. University of Tasmania, UTAS1	New Caledonia	LC405276	LC404731	LC404862	LC405404	LC405143
	041, cult. University of Tasmania, UTAS2	New Caledonia	LC405280	LC404736	LC404867	LC405409	LC405148
C. oblonga Rich. subsp. *oblonga*	130, TAS1-1	Tas	LC405357	LC404815	LC404955	LC405498	LC405229
	131, TAS1-2	Tas	LC405358	LC404816	LC404956	LC405499	LC405230
C. oblonga subsp. *corangensis* K.D.Hill	078, Corang River 1, -35.204, 150.051	NSW	LC405307	LC404765	LC404904	LC405446	LC405177
	079, Corang River 2, -35.204, 150.051	NSW	LC405308	LC404766	LC404905	LC405447	LC405178
C. oblonga Rich. subsp. *parva* K.D.Hill	127, Crisp 11285	NSW	LC405354	LC404812	LC404952	LC405495	LC405226
	128, Crisp 11286	NSW	LC405355	LC404813	LC404953	LC405496	LC405227
C. pancheri (Carrière) Byng	037, cult. University of Tasmania, UTAS1	New Caledonia	LC405277	LC404732	LC404863	LC405405	LC405144
	046, cult. University of	New Caledonia	LC405285	LC404741	LC404872	LC405414	LC405153

	Tasmania, UTAS2						
C. preissii Miq.	119, Crisp 10025	WA	LC405346	LC404804	LC404944	LC405487	LC405218
	115, Crisp 11016	WA	LC405342	LC404800	LC404940	LC405483	LC405214
C. pyramidalis (Miq.) J.E.Piggin & J.J.Bruhl	022, Crisp 10009	WA	LC405263	LC404718	LC404849	LC405391	LC405130
	112, Crisp 10881	WA	LC405339	LC404797	LC404937	LC405480	LC405480
C. rhomboidea R.Br. ex Rich. & A.Rich.	086, L. Prior, Blue Mts, tree A	NSW	LC405315	LC404773	LC404912	LC405454	LC405185
	034, cult. University of Tasmania, UTAS1		LC405274	LC404729	LC404860	LC405402	LC405141
C. roei (Endl.) F.Muell.	018, Crisp 9922	WA	LC405259	LC404714	LC404845	LC405387	LC405126
	116, Crisp 11040	WA	LC405343	LC404801	LC404941	LC405484	LC405215
C. sulcata (Parl.) Schltr.	035, cult. University of Tasmania, UTAS1	New Caledonia	LC405275	LC404730	LC404861	LC405403	LC405142
	040, cult. University of Tasmania, UTAS2	New Caledonia	LC405279	LC404735	LC404866	LC405408	LC405147
C. tuberculata R.Br. ex R.T.Baker & H.G.Sm.	020, Crisp 9975	WA	LC405261	LC404716	LC404847	LC405389	LC405128
C. verrucosa (A.Cunn. ex Endl.) R.Br. ex Mirb.	021, Crisp 9883	SA	LC405262	LC404717	LC404848	LC405390	LC405129
	123, L. Prior, Calder Hwy 5	Vic	LC405350	LC404808	LC404948	LC405491	LC405222
Diselma archeri Hook.f.	042, cult. University of Tasmania, UTAS1	Tas	LC405281	LC404737	LC404868	LC405410	LC405149
Fitzroya cupressoides (Molina) I.M.Johnst.	043, cult. University of Tasmania, UTAS1	Patagonia	LC405282	LC404738	LC404869	LC405411	LC405150
	094, Crisp 10673	Argentina	LC405322	LC404780	LC404920	LC405462	LC405193
Libocedrus austrocaledonica Brongn. & Gris	107, cult. Royal Tasmanian Botanic Garden	New Caledonia	LC405334	LC404792	LC404932	LC405475	LC405206
L. bidwillii Hook.f.	106, cult. Royal Tasmanian Botanic Garden	New Zealand	LC405333	LC404791	LC404931	LC405474	LC405474
L. chevalieri J.Buchholz	108, cult. Royal Tasmanian Botanic Garden	New Caledonia	LC405335	LC404793	LC404933	LC405476	LC405207
L. yateensis Guillaumin	045, cult. University of Tasmania, UTAS1	New Caledonia	LC405284	LC404740	LC404871	LC405413	LC405152
Papuacedrus papuana (F. Muell.) H.L. Li	047, cult. University of Tasmania, UTAS1	New Guinea	LC405286	LC404742	LC404873	LC405415	LC405154

Pilgerodendron uviferum (D. Don) Florin	048, cult. University of Tasmania, UTAS1	Argentina	LC405287	LC404743	LC404874	LC405416	LC405155
	093, Crisp 10672	Argentina	LC405321	LC404779	LC404919	LC405461	LC405192
Widdringtonia cedarbergensis J.A.Marsh	049, cult. University of Tasmania, UTAS1	South Africa	LC405288	LC404744	LC404875	LC405417	LC405156
W. schwarzii (Marloth) Mast.	050, cult. University of Tasmania, UTAS1	South Africa	LC405289	LC404745	LC404876	LC405418	LC405157
**Cupressaceae subfam. Cupressoideae**							
Calocedrus decurrens (Torr.) Florin	096, Crisp 10686	USA, Mexico	LC405324	LC404782	LC404922	LC405464	LC405195
C. formosana (Florin) Florin	053, cult. Kyoto University	Taiwan	LC405290	LC404746	LC404879	LC405421	LC405158
Chamaecyparis obtusa (Siebold & Zucc.) Endl.	056, cult. Kyoto University	Japan	LC405292	LC404748	LC404882	LC405424	LC405160
C. pisifera (Siebold & Zucc.) Endl.	055, cult. Kyoto University	Japan	LC405291	LC404747	LC404881	LC405423	LC405159
Cupressus cashmeriana Royle ex Carr.	097, Crisp 10687	Himalayas	LC405325	LC404783	LC404923	LC405465	LC405196
C. funebris Endl.	059, cult. Kyoto University	China, Vietnam	LC405295	LC404751	LC404885	LC405427	LC405163
C. sempervirens L.	060, cult. Kyoto University	Mediterranean	LC405296	LC404752	LC404886	LC405428	LC405164
C. torulosa D.Don ex Lamb.	102, Crisp 10692	Himalayas, China, Vietnam	LC405329	LC404787	LC404927	LC405470	LC405201
Hesperocyparis arizonica (Greene) Bartel	099, Crisp 10689	USA, Mexico	LC405326	LC404784	LC404925	LC405467	LC405198
H. macrocarpa (Hartw. ex Gordon) Bartel	100, Crisp 10690	California	LC405327	LC404785	LC404926	LC405468	LC405199
Juniperus chinensis L.	061, cult. Kyoto University	East Asia	LC405297	LC404753	LC404887	LC405429	LC405165
J. chinensis var. *sargentii* A.Henry	063, cult. Kyoto University	Japan	LC405299	LC404755	LC404889	LC405431	LC405167
J. deppeana Steud.	103, Crisp 10693	USA, Mexico	LC405330	LC404788	LC404928	LC405471	LC405202
J. rigida Siebold & Zucc.	062, cult. Kyoto University	East Asia	LC405298	LC404754	LC404888	LC405430	LC405166
Platycladus orientalis (L.) Franco	069, cult. Kyoto University	East Asia	LC405305	LC404761	LC404895	LC405437	LC405173
Tetraclinis articulata (Vahl) Mast.	104, cult. UC Davis	Mediterranean	LC405331	LC404789	LC404929	LC405472	LC405203

	105, cult. UC Davis	Mediterranean	LC405332	LC404790	LC404930	LC405473	LC405204
Thuja occidentalis L.	071, cult. Kyoto University	Canada, USA	LC405306	LC404763	LC404897	LC405439	LC405175
**Other Cupressaceae subfamilies**							
Athrotaxis selaginoides D.Don	109, Mt Read	Tas	LC405336	LC404794	LC404934	LC405477	LC405208
Cryptomeria japonica (Thunb. ex L.f.) D.Don	057, cult. Kyoto University	China, Japan	LC405293	LC404749	LC404883	LC405425	LC405161
Cunninghamia lanceolata (Lamb.) Hook.	058, cult. Kyoto University	SE Asia	LC405294	LC404750	LC404884	LC405426	LC405162
Metasequoia glyptostroboides H.H.Hu & W.C.Cheng	064, cult. Kyoto University	China	LC405300	LC404756	LC404890	LC405432	LC405168
Sequoia sempervirens (D. Don) Endl.	065, cult. Kyoto University	Western USA	LC405301	LC404757	LC404891	LC405433	LC405169
Sequoiadendron giganteum (Lindl.) J. Buchholz	095, Crisp 10685	California	LC405323	LC404781	LC404921	LC405463	LC405194
Taiwania cryptomeroides Hayata	066, cult. Kyoto University	Vietnam, Taiwan	LC405302	LC404758	LC404892	LC405434	LC405170
Taxodium distichum (L.) Rich.	068, cult. Kyoto University	SE USA	LC405304	LC404760	LC404894	LC405436	LC405172
T. mucronatum Ten.	067, cult. Kyoto University	Mexico, Guatemala	LC405303	LC404759	LC404893	LC405435	LC405171

Part 2: Chloroplast DNA sequences (cont.)

Taxon	Accession number, voucher or source (Herbarium)	*Geographic origin*	trnL–trnF	trnH–psbA	petG-trnP	trnC–trnD
**Cupressaceae subfam. Callitroideae**						
Austrocedrus chilensis (D.Don) Florin & Boutelje	089, Crisp 10667	Argentina	LC405875	LC405736	LC405055	LC405597
	090, Crisp 10669	Argentina	LC405876	LC405737	LC405056	LC405598
Callitris acuminata (Parl.) F.Muell.	113, Crisp 10908	WA	LC405899	LC405760	LC405079	LC405620
	124, Crisp 11186	WA	LC405910	LC405771	LC405090	LC405631
C. arenaria (C.A.Gardner) J.E.Piggin & J.J.Bruhl	111, Crisp 10803	WA	LC405897	LC405758	LC405077	LC405618
	125, Crisp 11187	WA	LC405911	LC405772	LC405091	LC405632

C. baileyi C.T.White	132, Crisp 11289A	Qld	LC405918	LC405779	LC405098	LC405639
	001, Crisp 10153A	Qld	LC405791	LC405651	LC404969	LC405512
C. canescens (Parl.) S.T.Blake	002, Crisp 10118	SA	LC405792	LC405652	LC404970	LC405513
	135, Crisp 9703	WA	LC405921	LC405782	LC405101	LC405642
C. columellaris F.Muell.	004, Crisp 10321	Qld	LC405794	LC405654	LC404972	LC405515
	084, Crisp 10703	NSW	LC405870	LC405731	LC405050	LC405592
C. drummondii (Parl.) Benth. & Hook.f. ex F.Muell.	114, Crisp 10998	WA	LC405900	LC405761	LC405080	LC405621
	005, Crisp 9945	WA	LC405795	LC405655	LC404973	LC405516
C. endlicheri (Parl.) F.M.Bailey	007, Crisp 10345	Qld	LC405797	LC405657	LC404975	LC405518
	016, Crisp 10340	Qld	LC405806	LC405666	LC404984	LC405527
C. glaucophylla Joy Thomps. & L.A.S.Johnson	025, Crisp 9796	NSW	LC405813	LC405674	LC404991	LC405534
	136, Central Australia, CA1-1 (Sakaguchi *et al.*, 2013)	NT	LC405922	LC405783	LC405102	LC405643
	142, WA3-1 (Sakaguchi *et al.*, 2013)	WA	LC405928	LC405789	LC405108	LC405649
C. gracilis R.T.Baker	026, Crisp 9801	SA	LC405814	LC405675	LC404992	LC405535
	019, Crisp 9894	WA	LC405808	LC405668	LC404986	LC405529
C. intratropica R.T.Baker & H.G.Sm.	028, Crisp 9760	NT	LC405816	LC405677	LC404994	LC405537
	011, Crisp 10221	Qld	LC405801	LC405661	LC404979	LC405522
C. macleayana (F.Muell.) F.Muell.	014, Crisp 10138	NSW	LC405804	LC405664	LC404982	LC405525
	080, Mt Windsor 2, -16.28, 145.08	Qld	LC405866	LC405727	LC405046	LC405588
C. monticola J.Garden	085, Crisp 10707	NSW	LC405871	LC405732	LC405051	LC405593
	015, Fethers 42 (CANB)		LC405805	LC405665	LC404983	LC405526
C. muelleri (Parl.) Benth. & Hook.f. ex F.Muell.	033, Beesley 281 (CANB)	NSW	LC405821	LC405682	LC404999	LC405542
	087, Blue Mts B (Sakaguchi *et al.*, 2013)	NSW	LC405873	LC405734	LC405053	LC405595
C. neocaledonica Dümmer	036, cult. University of Tasmania, UTAS1	New Caledonia	LC405824	LC405685	LC405002	LC405545
	041, cult. University of Tasmania, UTAS2	New Caledonia	LC405829	LC405690	LC405007	LC405550
C. oblonga Rich. subsp. *oblonga*	130, TAS1-1	Tas	LC405916	LC405777	LC405096	LC405637
	131, TAS1-2	Tas	LC405917	LC405778	LC405097	LC405638
C. oblonga subsp. *corangensis*	078, Corang River 1, -	NSW	LC405864	LC405725	LC405044	LC405586

K.D.Hill	35.204, 150.051					
	079, Corang River 2, -35.204, 150.051	NSW	LC405865	LC405726	LC405045	LC405587
C. oblonga Rich. subsp. *parva* K.D.Hill	127, Crisp 11285	NSW	LC405913	LC405774	LC405093	LC405634
	128, Crisp 11286	NSW	LC405914	LC405775	LC405094	LC405635
C. pancheri (Carrière) Byng	037, cult. University of Tasmania, UTAS1	New Caledonia	LC405825	LC405686	LC405003	LC405546
	046, cult. University of Tasmania, UTAS2	New Caledonia	LC405834	LC405695	LC405012	LC405555
C. preissii Miq.	119, Crisp 10025	WA	LC405905	LC405766	LC405085	LC405626
	115, Crisp 11016	WA	LC405901	LC405762	LC405081	LC405622
C. pyramidalis (Miq.) J.E.Piggin & J.J.Bruhl	022, Crisp 10009	WA	LC405811	LC405671	LC404989	LC405532
	112, Crisp 10881	WA	LC405898	LC405759	LC405078	LC405619
C. rhomboidea R.Br. ex Rich. & A.Rich.	086, L. Prior, Blue Mts, tree A	NSW	LC405872	LC405733	LC405052	LC405594
	034, cult. University of Tasmania, UTAS1		LC405822	LC405683	LC405000	LC405543
C. roei (Endl.) F.Muell.	018, Crisp 9922	WA	LC405807	LC405667	LC404985	LC405528
	116, Crisp 11040	WA	LC405902	LC405763	LC405082	LC405623
C. sulcata (Parl.) Schltr.	035, cult. University of Tasmania, UTAS1	New Caledonia	LC405823	LC405684	LC405001	LC405544
	040, cult. University of Tasmania, UTAS2	New Caledonia	LC405828	LC405689	LC405006	LC405549
C. tuberculata R.Br. ex R.T.Baker & H.G.Sm.	020, Crisp 9975	WA	LC405809	LC405669	LC404987	LC405530
C. verrucosa (A.Cunn. ex Endl.) R.Br. ex Mirb.	021, Crisp 9883	SA	LC405810	LC405670	LC404988	LC405531
	123, L. Prior, Calder Hwy 5	Vic	LC405909	LC405770	LC405089	LC405630
Diselma archeri Hook.f.	042, cult. University of Tasmania, UTAS1	Tas	LC405830	LC405691	LC405008	LC405551
Fitzroya cupressoides (Molina) I.M.Johnst.	043, cult. University of Tasmania, UTAS1	Patagonia	LC405831	LC405692	LC405009	LC405552
	094, Crisp 10673	Argentina	LC405880	LC405741	LC405060	LC405601
Libocedrus austrocaledonica Brongn. & Gris	107, cult. Royal Tasmanian Botanic Garden	New Caledonia	LC405893	LC405754	LC405073	LC405614

L. bidwillii Hook.f.	106, cult. Royal Tasmanian Botanic Garden	New Zealand	LC405892	LC405753	LC405072	LC405613
L. chevalieri J.Buchholz	108, cult. Royal Tasmanian Botanic Garden	New Caledonia	LC405894	LC405755	LC405074	LC405615
L. yateensis Guillaumin	045, cult. University of Tasmania, UTAS1	New Caledonia	LC405833	LC405694	LC405011	LC405554
Papuacedrus papuana (F. Muell.) H.L. Li	047, cult. University of Tasmania, UTAS1	New Guinea	LC405835	LC405696	LC405013	LC405556
Pilgerodendron uviferum (D. Don) Florin	048, cult. University of Tasmania, UTAS1	Argentina	LC405836	LC405697	LC405014	LC405557
	093, Crisp 10672	Argentina	LC405879	LC405740	LC405059	LC405600
Widdringtonia cedarbergensis J.A.Marsh	049, cult. University of Tasmania, UTAS1	South Africa	LC405837	LC405698	LC405015	LC405558
W. schwarzii (Marloth) Mast.	050, cult. University of Tasmania, UTAS1	South Africa	LC405838	LC405699	LC405016	LC405559
**Cupressaceae subfam. Cupressoideae**						
Calocedrus decurrens (Torr.) Florin	096, Crisp 10686	USA, Mexico	LC405882	LC405743	LC405062	LC405603
C. formosana (Florin) Florin	053, cult. Kyoto University	Taiwan	LC405841	LC405702	LC405019	LC405562
Chamaecyparis obtusa (Siebold & Zucc.) Endl.	056, cult. Kyoto University	Japan	LC405844	LC405705	LC405022	LC405565
C. pisifera (Siebold & Zucc.) Endl.	055, cult. Kyoto University	Japan	LC405843	LC405704	LC405021	LC405564
Cupressus cashmeriana Royle ex Carr.	097, Crisp 10687	Himalayas	LC405883	LC405744	LC405063	LC405604
C. funebris Endl.	059, cult. Kyoto University	China, Vietnam	LC405847	LC405708	LC405025	LC405568
C. sempervirens L.	060, cult. Kyoto University	Mediterranean	LC405848	LC405709	LC405026	LC405569
C. torulosa D.Don ex Lamb.	102, Crisp 10692	Himalayas, China, Vietnam	LC405888	LC405749	LC405068	LC405609
Hesperocyparis arizonica (Greene) Bartel	099, Crisp 10689	USA, Mexico	LC405885	LC405746	LC405065	LC405606
H. macrocarpa (Hartw. ex Gordon) Bartel	100, Crisp 10690	California	LC405886	LC405747	LC405066	LC405607
Juniperus chinensis L.	061, cult. Kyoto University	East Asia	LC405849	LC405710	LC405027	LC405570
J. chinensis var. *sargentii* A.Henry	063, cult. Kyoto University	Japan	LC405851	LC405712	LC405029	LC405572

J. deppeana Steud.	103, Crisp 10693	USA, Mexico	LC405889	LC405750	LC405069	LC405610
J. rigida Siebold & Zucc.	062, cult. Kyoto University	East Asia	LC405850	LC405711	LC405028	LC405571
Platycladus orientalis (L.) Franco	069, cult. Kyoto University	East Asia	LC405857	LC405718	LC405035	LC405578
Tetraclinis articulata (Vahl) Mast.	104, cult. UC Davis	Mediterranean	LC405890	LC405751	LC405070	LC405611
	105, cult. UC Davis	Mediterranean	LC405891	LC405752	LC405071	LC405612
Thuja occidentalis L.	071, cult. Kyoto University	Canada, USA	LC405858	LC405720	LC405037	LC405580
**Other Cupressaceae subfamilies**						
Athrotaxis selaginoides D.Don	109, Mt Read	Tas	LC405895	LC405756	LC405075	LC405616
Cryptomeria japonica (Thunb. ex L.f.) D.Don	057, cult. Kyoto University	China, Japan	LC405845	LC405706	LC405023	LC405566
Cunninghamia lanceolata (Lamb.) Hook.	058, cult. Kyoto University	SE Asia	LC405846	LC405707	LC405024	LC405567
Metasequoia glyptostroboides H.H.Hu & W.C.Cheng	064, cult. Kyoto University	China	LC405852	LC405713	LC405030	LC405573
Sequoia sempervirens (D. Don) Endl.	065, cult. Kyoto University	Western USA	LC405853	LC405714	LC405031	LC405574
Sequoiadendron giganteum (Lindl.) J. Buchholz	095, Crisp 10685	California	LC405881	LC405742	LC405061	LC405602
Taiwania cryptomeroides Hayata	066, cult. Kyoto University	Vietnam, Taiwan	LC405854	LC405715	LC405032	LC405575
Taxodium distichum (L.) Rich.	068, cult. Kyoto University	SE USA	LC405856	LC405717	LC405034	LC405577
T. mucronatum Ten.	067, cult. Kyoto University	Mexico, Guatemala	LC405855	LC405716	LC405033	LC405576

Part 3: Nuclear DNA sequences

Taxon	Accession number, voucher or source (Herbarium)	*Geographic origin*	c257	c22306	c36749
**Cupressaceae subfam. Callitroideae**					
Austrocedrus chilensis (D.Don) Florin & Boutelje	089, Crisp 10667	Argentina	LC404568	LC404474	LC404661

	090, Crisp 10669	Argentina	LC404569	LC404475	-
Callitris acuminata (Parl.) F.Muell.	113, Crisp 10908	WA	LC404579	-	-
	124, Crisp 11186	WA	LC404590	-	LC404678
C. arenaria (C.A.Gardner) J.E.Piggin & J.J.Bruhl	111, Crisp 10803	WA	LC404577	LC404482	LC404667
	125, Crisp 11187	WA	LC404591	LC404493	LC404679
C. baileyi C.T.White	132, Crisp 11289A	Qld	LC404598	LC404500	LC404686
	001, Crisp 10153A	Qld	LC404511	LC404416	LC404610
C. canescens (Parl.) S.T.Blake	002, Crisp 10118	SA	LC404512	LC404417	LC404611
	135, Crisp 9703	WA	LC404601	LC404503	LC404689
C. columellaris F.Muell.	004, Crisp 10321	Qld	LC404514	LC404419	LC404613
	084, Crisp 10703	NSW	LC404563	LC404469	LC404656
C. drummondii (Parl.) Benth. & Hook.f. ex F.Muell.	114, Crisp 10998	WA	LC404580	LC404484	-
	005, Crisp 9945	WA	LC404515	LC404420	LC404614
C. endlicheri (Parl.) F.M.Bailey	007, Crisp 10345	Qld	-	LC404422	LC404616
	016, Crisp 10340	Qld	-	LC404431	LC404625
C. glaucophylla Joy Thomps. & L.A.S.Johnson	025, Crisp 9796	NSW	LC404532	LC404440	LC404633
	136, Central Australia, CA1-1 (Sakaguchi *et al.*, 2013)	NT	LC404602	-	LC404690
	142, WA3-1 (Sakaguchi *et al.*, 2013)	WA	LC404608	LC404509	LC404696
C. gracilis R.T.Baker	026, Crisp 9801	SA	LC404533	LC404441	LC404634
	019, Crisp 9894	WA	LC404526	LC404434	LC404627
C. intratropica R.T.Baker & H.G.Sm.	028, Crisp 9760	NT	LC404535	LC404443	LC404636
	011, Crisp 10221	Qld	LC404519	LC404426	LC404620
C. macleayana (F.Muell.) F.Muell.	014, Crisp 10138	NSW	LC404522	LC404429	LC404623
	080, Mt Windsor 2, -16.28, 145.08	Qld	LC404559	LC404465	LC404652
C. monticola J.Garden	085, Crisp 10707	NSW	LC404564	LC404470	LC404657
	015, Fethers 42 (CANB)		LC404523	LC404430	LC404624
C. muelleri (Parl.) Benth. & Hook.f. ex F.Muell.	033, Beesley 281 (CANB)	NSW	LC404540	LC404448	LC404641
	087, Blue Mts B (Sakaguchi *et al.*, 2013)	NSW	LC404566	LC404472	LC404659

C. neocaledonica Dümmer	036, cult. University of Tasmania, UTAS1	New Caledonia	LC404543	LC404451	LC404644
	041, cult. University of Tasmania, UTAS2	New Caledonia	LC404547	LC404455	LC404648
C. oblonga Rich. subsp. *oblonga*	130, TAS1-1	Tas	LC404596	LC404498	LC404684
	131, TAS1-2	Tas	LC404597	LC404499	LC404685
C. oblonga subsp. *corangensis* K.D.Hill	078, Corang River 1, -35.204, 150.051	NSW	LC404557	LC404463	LC404650
	079, Corang River 2, -35.204, 150.051	NSW	LC404558	LC404464	LC404651
C. oblonga Rich. subsp. *parva* K.D.Hill	127, Crisp 11285	NSW	LC404593	LC404495	LC404681
	128, Crisp 11286	NSW	LC404594	LC404496	LC404682
C. pancheri (Carrière) Byng	037, cult. University of Tasmania, UTAS1	New Caledonia	LC404544	LC404452	LC404645
	046, cult. University of Tasmania, UTAS2	New Caledonia	LC404552	LC404460	LC404649
C. preissii Miq.	119, Crisp 10025	WA	LC404585	LC404488	LC404673
	115, Crisp 11016	WA	LC404581	LC404485	LC404669
C. pyramidalis (Miq.) J.E.Piggin & J.J.Bruhl	022, Crisp 10009	WA	LC404529	LC404437	LC404630
	112, Crisp 10881	WA	LC404578	LC404483	LC404668
C. rhomboidea R.Br. ex Rich. & A.Rich.	086, L. Prior, Blue Mts, tree A	NSW	LC404565	LC404471	LC404658
	034, cult. University of Tasmania, UTAS1		LC404541	LC404449	LC404642
C. roei (Endl.) F.Muell.	018, Crisp 9922	WA	LC404525	LC404433	LC404626
	116, Crisp 11040	WA	LC404582		LC404670
C. sulcata (Parl.) Schltr.	035, cult. University of Tasmania, UTAS1	New Caledonia	LC404542	LC404450	LC404643
	040, cult. University of Tasmania, UTAS2	New Caledonia	LC404546	LC404454	LC404647
C. tuberculata R.Br. ex R.T.Baker & H.G.Sm.	020, Crisp 9975	WA	LC404527	LC404435	LC404628
C. verrucosa (A.Cunn. ex Endl.) R.Br. ex Mirb.	021, Crisp 9883	SA	LC404528	LC404436	LC404629
	123, L. Prior, Calder Hwy 5	Vic	LC404589	LC404492	LC404677

Diselma archeri Hook.f.	042, cult. University of Tasmania, UTAS1	Tas	LC404548	LC404456	-
Fitzroya cupressoides (Molina) I.M.Johnst.	043, cult. University of Tasmania, UTAS1	Patagonia	LC404549	LC404457	-
	094, Crisp 10673	Argentina	LC404572	LC404478	-
Libocedrus austrocaledonica Brongn. & Gris	107, cult. Royal Tasmanian Botanic Garden	New Caledonia	LC404574	LC404479	LC404663
L. bidwillii Hook.f.	106, cult. Royal Tasmanian Botanic Garden	New Zealand	LC404573	-	LC404662
L. chevalieri J.Buchholz	108, cult. Royal Tasmanian Botanic Garden	New Caledonia	LC404575	LC404480	LC404664
L. yateensis Guillaumin	045, cult. University of Tasmania, UTAS1	New Caledonia	LC404551	LC404459	-
Papuacedrus papuana (F. Muell.) H.L. Li	047, cult. University of Tasmania, UTAS1	New Guinea	LC404553	-	-
Pilgerodendron uviferum (D. Don) Florin	048, cult. University of Tasmania, UTAS1	Argentina	LC404554	LC404461	-
	093, Crisp 10672	Argentina	LC404571	LC404477	-
Widdringtonia cedarbergensis J.A.Marsh	049, cult. University of Tasmania, UTAS1	South Africa	LC404555	-	-
W. schwarzii (Marloth) Mast.	050, cult. University of Tasmania, UTAS1	South Africa	LC404556	LC404462	-
**Cupressaceae subfam. Cupressoideae**					
Calocedrus decurrens (Torr.) Florin	096, Crisp 10686	USA, Mexico	-	-	-
C. formosana (Florin) Florin	053, cult. Kyoto University	Taiwan	-	-	-
Chamaecyparis obtusa (Siebold & Zucc.) Endl.	056, cult. Kyoto University	Japan	-	-	-
C. pisifera (Siebold & Zucc.) Endl.	055, cult. Kyoto University	Japan	-	-	-
Cupressus cashmeriana Royle ex Carr.	097, Crisp 10687	Himalayas	-	-	-
C. funebris Endl.	059, cult. Kyoto University	China, Vietnam	-	-	-
C. sempervirens L.	060, cult. Kyoto University	Mediterranean	-	-	-
C. torulosa D.Don ex Lamb.	102, Crisp 10692	Himalayas, China, Vietnam	-	-	-

Hesperocyparis arizonica (Greene) Bartel	099, Crisp 10689	USA, Mexico	-	-	-
H. macrocarpa (Hartw. ex Gordon) Bartel	100, Crisp 10690	California	-	-	-
Juniperus chinensis L.	061, cult. Kyoto University	East Asia	-	-	-
J. chinensis var. *sargentii* A.Henry	063, cult. Kyoto University	Japan	-	-	-
J. deppeana Steud.	103, Crisp 10693	USA, Mexico	-	-	-
J. rigida Siebold & Zucc.	062, cult. Kyoto University	East Asia	-	-	-
Platycladus orientalis (L.) Franco	069, cult. Kyoto University	East Asia	-	-	-
Tetraclinis articulata (Vahl) Mast.	104, cult. UC Davis	Mediterranean	-	-	-
	105, cult. UC Davis	Mediterranean	-	-	-
Thuja occidentalis L.	071, cult. Kyoto University	Canada, USA	-	-	-
**Other Cupressaceae subfamilies**					
Athrotaxis selaginoides D.Don	109, Mt Read	Tas	-	-	LC404665
Cryptomeria japonica (Thunb. ex L.f.) D.Don	057, cult. Kyoto University	China, Japan	-	-	-
Cunninghamia lanceolata (Lamb.) Hook.	058, cult. Kyoto University	SE Asia	-	-	-
Metasequoia glyptostroboides H.H.Hu & W.C.Cheng	064, cult. Kyoto University	China	-	-	-
Sequoia sempervirens (D. Don) Endl.	065, cult. Kyoto University	Western USA	-	-	-
Sequoiadendron giganteum (Lindl.) J. Buchholz	095, Crisp 10685	California	-	-	-
Taiwania cryptomeroides Hayata	066, cult. Kyoto University	Vietnam, Taiwan	-	-	-
Taxodium distichum (L.) Rich.	068, cult. Kyoto University	SE USA	-	-	-
T. mucronatum Ten.	067, cult. Kyoto University	Mexico, Guatemala	-	-	-

Table S2 Loci sequenced, primers used for PCR and their design sources.

Locus<br>Chloroplast DNA		Primer code	Primer sequence	Source
rbcL gene	F:	rbcL-F	ATGTCACCACAAACAGAAACTAAAGCAAGT	(Little *et al.*, 2004)
	R:	rbcL-R	TCACAAGCAGCAGCTAGTTCAGGACTC	(Little *et al.*, 2004)
matK gene	F:	matK-F	CCAAATTCGTTCTCTCTGTG	(Kusumi *et al.*, 2000)
	R:	matK-R	TATTCCATGAGTCAGGAGAG	(Kusumi *et al.*, 2000)
matK–trnK	F:	Ivy5-matK6	CCATGATCATGAGCAAGTGC	(Grivet & Petit, 2002)
	R:	Ivy5-K1K2-trnK24	CAACGGTAGAGTACTCGGCTTTTA	(Grivet & Petit, 2002)
rpl16	F:	rpL16F71	GCTATGCTTAGTGTGTGACTCGTTG	(Shaw *et al.*, 2005)
	R:	rpL16R1516	CCCTTCATTCTTCCTCTATGTTG	(Shaw *et al.*, 2005)
psbH–psbB	F:	psbB	TCAAYRGTYTGTGTAGCCAT	(Shaw *et al.*, 2005)
	R:	psbH	TCCAAAAANKKGGAGATCCAAC	(Shaw *et al.*, 2005)
trnL–trnF	F:	TabC	CGAAATCGGTAGACGCTACG	(Shaw *et al.*, 2005)
	R:	TabF	ATTTGAACTGGTGACACGAG	(Shaw *et al.*, 2005)
trnH–psbA	F:	$trnH^{GUG}$	CGCGCATGGTGGATTCACAATCC	(Shaw *et al.*, 2005)
	R:	psbA	GTTATGCATGAACGTAATGCTC	(Shaw *et al.*, 2005)
petG-trnP	F:	petG	GGTCTAATTCCTATAACTTTGGC	(Huang *et al.*, 2002)
	R:	trnP	GGGATGTGGCGCAGCTTGG	(Huang *et al.*, 2002)
trnC–trnD	F:	trnC (tRNA-Cys(GCA))	CCAGTTCAAATCTGGGTGTC	(Lee & Wen, 2004)
	R:	petN 1R	CCCAAGCAAGACTTACTATATCC	(Lee & Wen, 2004)
Nuclear loci				
c257: inorganic pyrophosphatase	F:	c257-F	TCATCGGTTTGATTGTTGGA	Designed for this study
	R:	c257-R	CAGCCTTATGGGGATCTGAA	Designed for this study
c22306: erd1	F:	c22306-F	GTGATTCCCGACTCCAAAAA	Designed for this study

protein				
	R:	c22306-R	AGTCTGAACCTTCCGACCTG	Designed for this study
c36749: alpha-glucan protein synthase	F:	c36749-F	GAAAGGATATCCGCGAACAA	Designed for this study
	R:	c36749-R	TTCCTGCTCTGAGGTCGGTA	Designed for this study

Table S3 Molecular-clock calibrations. Units are in Ma. Abbreviations: M = mean; O = offset; N = normal distribution; SD = standard deviation; L = lower; U = upper. Placements on the tree are all expressed in terms of two taxa that diverge at that node, i.e. at the MRCA of the specified taxa.

Number (Fig. S3)	Name	Placement on tree	Lognormal priors set	Uniform priors set	Explanation (if needed) with citations
1	Root	*Cunninghamia*–*Taiwania*	Normal: M = 215.0, SD = 2.5	Normal: M = 215.0, SD = 2.5	Root calibration, given a normal prior, using mean and SD of estimates by Mao *et al*. (2012)
2	Athro_st	*Athrotaxis*–*Callitris*	O = 105.0, M = 2.0, SD = 1.0	L = 105.0; U = 220.	Mao *et al.* (2012). Upper limit in this and the following priors is equal to the mean rootheight plus two SDs.
3	Sequoi_cr		O = 92.8, M = 2.0, SD = 1.0	L = 92.8; U = 220.	Mao *et al.* (2012)
4	Taxodioid_cr		O = 111.0, M = 2.0, SD = 1.0	L = 111.0; U = 220.	Mao *et al.* (2012)
5	Callitroid-Cupressoid	Callitroideae–Cupressoideae	O = 93.5, M = 2.0, SD	L = 93.5; U = 220.	*Widdringtonia americana*, which likely belongs to one of these subfamilies (Stockey *et al.*, 2005).

			= 1.0		
6	Cupressoid_cr	*Thuja–Cupressus*	O = 83.0, M = 2.0, SD = 1.0	L = 83.0; U = 220.	The oldest fossil is the late Cretaceous '*Chamaecyparis*' *corpulenta* (McIver 1994; Kotyk *et al.,* 2003; Stockey *et al.,* 2005), now considered to combined features of *Cupressus* and *Thuja*. Therefore, it is a good candidate for placement at MRCA of these lineages, ie crown node of Cupresseae.
7	Calocedrus_st	*Calocedrus–Tetraclinis*	O = 47.0, M = 2.0, SD = 1.0	L = 47.0; U = 220.	*Tetraclinis* dates from the early Eocene (Stockey *et al.*, 2005).
8	Juniperus_cr		O = 34.0, M = 2.0, SD = 1.0	L = 34.0; U = 220.	Mao *et al.* (2012)
9	Callitroid_cr	*Papuacedrus* - *Callitris*	O = 62.0, M = 2.0, SD = 1.0	L = 62.0; U = 220.	*Libocedrus* sp. cf. *bidwillii* (Pole, 1998) and other fossils of Palaeocene age (Hill & Brodribb, 1999), (Whang & Hill, 1999), which have the distinctive foliage characters of *Papuacedrus*, *Austrocedrus* and *Libocedrus*.
10	"C. octothamna"	*Austrocedrus–Callitris* or *Libocedrus–Callitris*	O = 99.6, M = 2.0, SD = 1.0	NA	Peters (1985). Alternative placements (shown in Fig. S3), or exclusion, of this calibration were tested using Bayes factors.
11	Libocedrus_mesibovii	*Libocedrus–*	O = 24.0,	NA	Hill & Carpenter (1989). Interpretation and placement of

		Pilgerodendron	M = 2.0, SD = 1.0		this fossil are discussed in the text. Bayes Factors preferred exclusion of this calibration.
12	Fitzroya_st	*Fitzroya–Diselma*	O = 30.0, M = 2.0, SD = 1.0	L = 30.0; U = 220.	The oldest fossil is *F. acutifolia* (Hill & Paull, 2003).
13	Callitris_cr	*C. roei–C. glaucophylla*	O = 28.3, M = 2.0, SD = 1.0	L = 28.3; U = 220.	*C. leaensis*, foliage and cones from early Oligocene of Tasmania (Paull & Hill, 2010), placed at the crown node of the genus (Mao *et al.*, 2012)

Table S4 Model comparisons using Bayes factors calculated from marginal likelihoods in BEAST. For each test, parameters not under comparison are held constant. Models are preference-ranked by their marginal log likelihoods. Asterisks after the Bayes Factors (BF) indicate their interpretation according to Raftery (1995): * = "positive" (2.2 ⩽ BF < 6), ** = "strong" (6 ⩽ BF < 10) and *** = "very strong" (BF ⩾ 10) evidence favouring the model with the higher likelihood. Absence of an asterisk indicates no preference for either model (0 < BF < 2.2, "not worth more than a bare mention"). Results are shown for both Path (P) and Stepping Stone (SS) sampling.

**Question/comparison**	**Other settings held constant during comparison**	**Model 1 (preferred)**	**Model 2 (non-preferred)**	**Model 3 (least preferred)**	**Bayes Factor supporting best model**
Clock model	*"C. octothamna"* and *L.*	Uncorrelated	Random local	NA	PS = 17.6***,

	mesibovii calibrations omitted	lognormal	clocks		SS = 24.4***
Calibration priors: uniform versus lognormal distribution	*"C. octothamna"* and *L. mesibovii* calibrations omitted	Lognormal	Uniform	NA	PS = 29.7***, SS = 31.4***
Calibration 11 using *L. mesibovii* (24 Ma) at MRCA of *Libocedrus* and *Pilgerodendron*	*"C. octothamna"* calibration omitted	*L. mesibovii* excluded	Inclusion of *L. mesibovii*	NA	PS = 33***, SS = 600***
Calibration 10 using *"Callitris octothamna"* (99.6 Ma)	No monophyly constraint; *L. mesibovii* calibration omitted	Placement at MRCA of *Callitris* and *Austrocedrus*	Exclusion of calibration	Placement at *Callitris* stem node	1 vs 2: PS = 0.84, SS = 0.22. 1 vs 3: PS = 4.72*, SS = 5.2*

Table S5 Trait data: fossil morphology and fire-adaptive traits. Trait states as follows. Phyllotaxis: 0 = ternate, 1 = decussate, 2 = spiral, 3 = quadrate. Leaf dimorphy: 0 = monomorphic, 1 = dimorphic. Cone scale phyllotaxis: 0 = 2 x 3, 1 = decussate, 2 = 2 x 4. Serotiny: 0 = absent; 1 = present. Habitat fire-prone: 0 = no, 1 = yes. † indicates an extinct fossil species (C. leaensis). Characters were scored for this species from the detailed descriptions and photographic plates in Paull & Hill (2010).

Taxon label	Leaf phyllotaxis	Leaf dimorphy	Cone scale phyllotaxis	Serotiny	Habitat fire-prone
000 *Callitris leaensis*†	0	0	0	0	0
001 *Callitris baileyi*	0	0	0	0	1
002 *Callitris canescens*	0	0	0	1	1
004 *Callitris columellaris*	0	0	0	0	1
005 *Callitris drummondii*	0	0	0	1	1
007 *Callitris endlicheri*	0	0	0	0	1
011 *Callitris intratropica*	0	0	0	0	1
014 *Callitris macleayana*	0 & 3	0	0 & 2	0	1
015 *Callitris monticola*	0	0	0	1	1
016 *Callitris endlicheri*	0	0	0	1	1
018 *Callitris roei*	0	0	0	1	1
019 *Callitris gracilis*	0	0	0	0	1
020 *Callitris tuberculata*	0	0	0	1	1
021 *Callitris verrucosa*	0	0	0	1	1
022 *Callitris pyramidalis*	0	0	0	1	1
025 *Callitris glaucophylla*	0	0	0	0	1
026 *Callitris gracilis*	0	0	0	1	1
028 *Callitris intratropica*	0	0	0	0	1
033 *Callitris muelleri*	0	0	0	1	1
034 *Callitris rhomboidea*	0	0	0	1	1
035 *Callitris sulcata*	0	0	0	0	0
036 *Callitris neocaledonica*	0	0	0	0	0
037 *Callitris pancheri*	3	0	2	0	0

040 *Callitris sulcata*	0	0	0	0	1
041 *Callitris neocaledonica*	0	0	0	0	1
042 *Diselma archeri*	0 & 1	0	1	0	0
043 *Fitzroya cupressoides*	0	0	0	0	0
045 *Libocedrus yateensis*	1	1	1	0	0
046 *Callitris pancheri*	3	0	2	0	1
047 *Papuacedrus papuana*	1	1	1	0	0
048 *Pilgerodendron uviferum*	0 & 1	0	1	0	1
049 *Widdringtonia cedarbergensis*	1 & 2	0	0	1	1
050 *Widdringtonia schwarzii*	1 & 2	0	1	1	1
053 *Calocedrus formosana*	1	1	1	0	0
055 *Chamaecyparis pisifera* cv. *squarrosa*	1	1	1	0	0
056 *Chamaecyparis obtusa*	1	1	1	0	0
059 *Cupressus funebris*	1	0	1	0	0
060 *Cupressus sempervirens*	1	0	1	1	1
061 *Juniperus chinensis*	1	0	1	0	0
062 *Juniperus rigida*	0	0	0	0	1
063 *Juniperus chinensis* var *sargentii*	1	0	1	0	0
069 *Platycladus orientalis*	1	1	1	0	1
071 *Thuja occidentalis*	1	1	1	0	0
078 *Callitris oblonga corangensis*	0	0	0	1	1
079 *Callitris oblonga corangensis*	0	0	0	1	1
080 *Callitris macleayana*	0 & 3	0	0 & 2	0	1
084 *Callitris columellaris*	0	0	0	0	1
085 *Callitris monticola*	0	0	0	1	1
086 *Callitris rhomboidea*	0	0	0	1	1
087 *Callitris muelleri*	0	0	0	1	1
089 *Austrocedrus chilensis*	1	1	1	0	1
090 *Austrocedrus chilensis*	1	1	1	0	0
093 *Pilgerodendron uviferum*	0 & 1	0	1	0	0

094 *Fitzroya cupressoides*	0	0	0	0	0
096 *Calocedrus decurrens*	1	1	1	0	1
097 *Cupressus cashmeriana*	1	0	1	0	0
099 *Hesperocyparis arizonica*	1	0	1	1	1
100 *Hesperocyparis macrocarpa*	1	0	1	1	1
102 *Cupressus torulosa*	1	0	1	1	1
103 *Juniperus deppeana*	1	0	1	0	1
104 *Tetraclinis articulata*	1	1	1	1	1
105 *Tetraclinis articulata*	1	1	1	1	1
106 *Libocedrus bidwillii*	1	1	1	0	0
107 *Libocedrus austrocaledonica*	1	1	1	0	0
108 *Libocedrus chevalieri*	1	1	1	0	1
111 *Callitris arenaria*	0	0	0	1	1
112 *Callitris pyramidalis*	0	0	0	1	1
113 *Callitris acuminata*	0	0	0	1	1
114 *Callitris drummondii*	0	0	0	1	1
115 *Callitris preissii*	0	0	0	1	1
116 *Callitris roei*	0	0	0	1	1
119 *Callitris preissii*	0	0	0	1	1
123 *Callitris verrucosa*	0	0	0	1	1
124 *Callitris acuminata*	0	0	0	1	1
125 *Callitris arenaria*	0	0	0	1	1
127 *Callitris oblonga parva*	0	0	0	1	1
128 *Callitris oblonga parva*	0	0	0	1	1
130 *Callitris oblonga oblonga*	0	0	0	1	1
131 *Callitris oblonga oblonga*	0	0	0	1	1
132 *Callitris baileyi*	0	0	0	0	1
135 *Callitris canescens*	0	0	0	1	1
136 *Callitris glaucophylla*	0	0	0	0	1
142 *Callitris glaucophylla*	0	0	0	0	1

Methods S1 Further details on assessment of previously unused fossils for calibration.

1. *"Callitris octothamna"*

These seed cone fossils could greatly increase the age estimate of *Callitris*, being from Cretaceous (c. 99.6 Ma) sediments near Winton, Queensland. The original assignment of this fossil to *Callitris* (Peters, 1985) was based on similarities of the shapes and arrangement of the leaves and seed cone scales. We used parsimony and maximum likelihood (Mk1 model, Lewis, 2001) mapping of three morphological characters (phyllotaxis, leaf dimorphism and cone scale arrangement) to assess whether the fossils have unique defining characters (synapomorphies) for any lineages within Callitroideae, including *Callitris*.

(a). Phyllotaxis. Leaves on the cone peduncle of *"C. octothamna"* are arranged in alternating ternate whorls with free tips (Peters, 1985). This is a derived feature relative to other Cupressaceae and is present in all adult *Callitris s.l.* except the New Caledonian *Callitris*, which has leaves in whorls of four (Table 1). Paedomorphosis likely accounts for quadrate leaves in adult plants, such as *Callitris pancheri* and *C. macleayana* (Supporting Information Fig. S4), because quadrate phyllotaxis occurs in seedlings of all Cupressaceae, including *Callitris* (Farjon, 2005). Within Callitroideae, ternate phyllotaxis is also present variably (to spiral) in adult plants of *Fitzroya*, *Diselma* and *Callitris macleayana*. Decussate adult phyllotaxis is plesiomorphic (Supporting Information Fig. S4) and predominant in Cupressoideae and other Callitroideae (*Austrocedrus*, *Libocedrus* and *Papuacedrus*). However, phyllotaxis is polymorphic in some callitroids: decussate or ternate in *Pilgerodendron*, decussate or spiral in *Widdringtonia*, and decussate or ternate in *Diselma*. Ternate phyllotaxis appears to be independently derived in Cupressoids (some *Juniperus*, e.g. *J. rigida*). The combination of this character-state (i.e., alternating ternate whorls) with fusion of the leaf to the internode for most of its basal length (not mapped) is unique to *Callitris* (Piggin & Bruhl, 2010). *"Callitris octothamna"* does not exhibit this distinctive basal leaf fusion but the fossils do not include vegetative foliage, which can differ from the scale leaves that are inserted on the cone peduncle (Paull & Hill, 2010). Parsimony and ML mapping (Table 2, Supporting Information Fig. S4) indicates that the transition between the plesiomorphic (decussate) and derived (ternate) state for phyllotaxis in callitroids is reconstructed as occurring between node B and the *Callitris*

stem (node A), with either partial reversal or a parallel origin of ternate phyllotaxis in *Widdringtonia* and *Diselma*.

(b). Leaf dimorphism. The plesiomorphic state of this character in Callitroideae (Supporting Information Fig. S5) is leaves dimorphic and compressed into one plane with those on the face of the plane being smaller and ± appressed, while the alternate leaf-pairs are marginal, larger and spreading laterally (Farjon, 2005). In the apomorphic state, leaves are monomorphic, even when decussate, and not compressed into one plane (Farjon, 2005). The monomorphic state is synapomorphic for all extant *Callitris* + *Widdringtonia*, *Fitzroya* and *Diselma*, but occurs homoplasiously with separate origins in in *Pilgerodendron* and Cupressoideae (*Cupressus-Juniperus* clade) (Supporting Information Fig. S5, Table S5). The dimorphic state occurs throughout other callitroids (*Papuacedrus*, *Austrocedrus*, *Libocedrus* and *Austrocedrus*) and is also found in Cupressoideae (Supporting Information Fig. S5, Table S5). *"Callitris octothamna"* has the derived state, with monomorphic scale-leaves on the peduncles. Trait mapping (Table 2, Supporting Information Fig. S5) reconstructs the transition between the plesiomorphic (dimorphic) and derived (monomorphic) state for callitroid leaves between node B and the *Callitris* stem (node A), with a parallel origin of monomorphy in *Pilgerodendron*.

(c). Cone-scale arrangement. Cone-scale and vegetative phyllotaxis are generally congruent in species of Callitroideae and Cupressoideae (Supporting Information Table S5) with few exceptions, one of which is *"C. octothamna"*. The plesiomorphic cone-scale arrangement is decussate and predominates across Cupressoideae and Callitroideae, except in *Juniperus* and the CFDW clade. However, the number of decussate pairs of cone scales varies (Farjon, 2005; Jagel & Dörken, 2015a; Jagel & Dörken, 2015b). Nearly all *Callitris* species have the derived state of two whorls of three, crowded into in a single pseudo-whorl of six alternately large and small scales. Exceptionally, in paedomorphic species, *Callitris* has two whorls of four (like the leaves) and in *C. macleayana* cone scale phyllotaxis is either 2 x 3 or 2 x 4, being congruent with leaf phyllotaxis of the subtending branch. *"Callitris octothamna"* cones closely resemble those of the extant species, and were described by Peters (1985) as a single whorl of eight, but are more likely 2 x 4, as in *Callitris* and *C. macleayana*. Closer examination of the photographs of cones in Peters (1985: figs 346–348) indicates that the alternate, smaller cone scales form an

outer whorl. Cone-scales of *Papuacedrus*, *Pilgerodendron*, *Libocedrus* and *Austrocedrus* are strictly decussate, which is the plesiomorphic condition. The *Widdringtonia-Diselma-Fitzroya* clade, which is sister to *Callitris*, is a mixture of 2 x 3 and 2 x 2. Mapped on the tree (Supporting Information Fig. S6; Table 2), the transition to 2 x 3 could have occurred in the stem of the CFDW clade (node B) with reversals in *Widdringtonia* and *Diselma*, or in the stem of *Callitris* (node A), with an independent gain in *Diselma*.

Given the polymorphisms and homoplasy, cone-scale phyllotaxis is unhelpful in placing *"C. octothamna"*, which has the unusual arrangement of cone scales in two whorls of four. Elsewhere in Callitroideae, only *Callitris* and *C. macleayana* have 2 x 4 (a rare variant in the latter), probably reflecting retention of juvenile leaves in the adult plant. However, in contrast to all extant callitroids (including *Callitris* and *C. macleayana*), *"C. octothamna"* uniquely combines ternate adult leaf phyllotaxis with 2 x 4 cone-scale phyllotaxis on the same branchlet. In Cupressoideae too, cone-scale arrangement usually echoes that of leaf phyllotaxis, except that cone scales are decussate in some species of *Juniperus* that have ternate leaves (Jagel & Dörken, 2015a).

(d). Adult leaf-base adnation to stem. This occurs in varying degrees across the callitroids but adnation for > 70% of length is claimed to be a synapomorphy for *Callitris* (Piggin & Bruhl, 2010). However, as this character varies continuously, the 70% cut-off is arbitrary. In the former genus *Actinostrobus*, which is included within *Callitris*, adnation is developed to a lesser degree (55–65%) and thus reversed by the 70% criterion. Also, in the Oligocene fossil *C. leaensis* (calibration 13 in Supporting Information Fig. S3), the adult leaves vary in adnation from 0 to 66% (Paull & Hill, 2010). Adnation also varies continuously in adult foliage within individuals of cupressoids (e.g., *Cupressus* and *Juniperus*). These taxa have widely spaced leaves with lengthy adnation on main stems and crowded, non-adnate leaves on side branchlets and tips. We have not mapped adnation on the tree because it is a continuously varying and labile trait and not a useful cladistic character.

In conclusion, *"Callitris octothamna"* shares apomorphies with some taxa within the callitroids, including ternate leaf phyllotaxis, 2 x 4 cone-scale phyllotaxis and loss of leaf dimorphism. The transition between the plesiomorphic and derived state for each of these

three characters is reconstructed as occurring between node B (stem of the CFDW clade) and node A (the *Callitris* crown node) (Figs S4–S6, Table 3). Therefore, all three characters indicate placement of the fossil as a calibration at either node A or its parent (node B). We used Bayes Factors to choose the best node for this calibration (see main text). *“Callitris octothamna”* also has similarity to *Juniperus* in leaf phyllotaxis, leaf monomorphism, cone-scale arrangement and the contrasting phyllotaxis between leaves and cone-scales. However, in other characters, *“C. octothamna”* does not appear to be close to *Juniperus*, and these similarities are likely to be convergent in the cupressoid and callitroid clades.

2. *Libocedrus*

Libocedrus is extant only in New Zealand and New Caledonia, and fossils are known from south-eastern Australia and New Zealand. Its sister taxon, *Pilgerodendron*, has a single species endemic in Patagonia and no recognised fossils. *Libocedrus* fossils range in age from the Palaeocene (> 55 Ma) to the Oligo-Miocene boundary (c. 24 Ma) and, with a single exception, are strictly vegetative. The characters (both gross morphological and cuticular) used to assign these fossils to *Libocedrus* appear to be symplesiomorphic features of Callitroideae, found in *Papuacedrus*, *Austrocedrus* and *Pilgerodendron* (Hill & Carpenter, 1989; Hill & Brodribb, 1999; Farjon, 2005; Pole, 2007). Examples of symplesiomorphic leaf characters are decussate, coplanar phyllotaxis (Supporting Information Fig. S4) and dimorphy (Supporting Information Fig. S5).

Exceptionally, the Oligo–Miocene (c. 24 Ma) fossils from Little Rapid River in Tasmania (Hill & Carpenter, 1989) have calibration potential because they include a cone (*L. mesibovii*) and two taxa described from detached foliage (*L. morrisonii* and *L. jacksonii*). The cones of *Libocedrus* and *Pilgerodendron*, ‘show a close structural similarity’ (Jagel & Dörken, 2015b) but that of *L. mesibovii* is asymmetrical with one cone-scale in the lower pair being much smaller than the other (Hill & Carpenter, 1989). Examining online photos and a specimen (CANB 381111), we found that extant *Pilgerodendron* occasionally has unequal lower cone-scales. This asymmetry is otherwise unknown in extant callitroid cypresses, including *Libocedrus*. *Pilgerodendron* and *Libocedrus* share a synapomorphy of spinescent appendages, resembling

‘Viking’ helmet horns, near the cone-scale apices (Farjon, 2005). Spines are not actually present on the fossil of *L. mesibovii* (Hill & Carpenter, 1989) but there are scars that the authors surmised were remnants of detached spines. Associated with the *L. mesibovii* fossil were detached vegetative sprigs described as *L. morrisonii* and *L. jacksonii* (Hill & Carpenter, 1989: fig. 3). The authors considered that *L. mesibovii* and *L. jacksonii* might be from the same plant but concluded that they were distinct species. We consider the leaves of *L. mesibovii* to resemble those of *Pilgerodendron* as well as any extant *Libocedrus* species (cf. Farjon, 2005).

We conclude that *L. mesibovii* could represent *Pilgerodendron*, *Libocedrus*, a common ancestor of both, or their sister group. Accordingly, we used this fossil as a calibration at the MRCA of the two genera and tested its fit relative to other calibrations using Bayes Factors. Placing *L. mesibovii* one node lower, at the shared stem of *Libocedrus* and *Pilgerodendron*, has no effect on dating because that node is estimated to be c. 90 Ma in the absence of this c. 24 Ma calibration.

Notes S1 References for Supporting Information.

Byng JW. 2015. *The gymnosperms handbook: A practical guide to extant families and genera of the world*. Hertford, UK: Plant Gateway Ltd.

de Laubenfels DJ, Husby CE, Griffith MP. 2012. Further nomenclatural action for the Cypresses (Cupressaceae). *Novon* 22: 8-15. doi: http://dx.doi.org/10.3417/2010056

Farjon A. 2005. *A monograph of Cupressaceae and Sciadopitys*. Kew: Royal Botanic Gardens.

Grivet D, Petit RJ. 2002. Phylogeography of the common ivy (*Hedera* sp.) in Europe: genetic differentiation through space and time. *Molecular Ecology* **11**: 1351–1362.

Hill KD 1998. 'Gymnosperms'—the paraphyletic stem of seed plants. In: McCarthy PM ed. *Flora of Australia, vol. 48. Ferns, gymnosperms, and allied groups*. Melbourne: CSIRO Publishing, 505–526.

Hill RS, Brodribb TJ. 1999. Southern conifers in time and space. *Australian Journal of Botany* **47**: 639–696.

Hill RS, Carpenter RJ. 1989. Tertiary gymnosperms from Tasmania: Cupressaceae. *Alcheringa* **13**: 89–102.

Hill RS, Paull R. 2003. *Fitzroya* (Cupressaceae) macrofossils from Cenozoic sediments in Tasmania, Australia. *Review of Palaeobotany and Palynology* **126**: 145–152.

Huang SSF, Hwang SY, Lin TP. 2002. Spatial pattern of chloroplast DNA variation of *Cyclobalanopsis glauca* in Taiwan and east Asia. *Molecular Ecology* **11**: 2349–2358.

Jagel A, Dörken VM. 2015a. Morphology and morphogenesis of the seed cones of the Cupressaceae—part II Cupressoideae. *Bulletin CCP* **4**: 51–78.

Jagel A, Dörken VM. 2015b. Morphology and morphogenesis of the seed cones of the Cupressaceae—part III: Callitroideae. *Bulletin CCP* **4**: 91–108.

Kass RE, Raftery AE. 1995. Bayes factors. *Journal of the American Statistical Association* **90**: 773–795.

Kotyk MEA, Basinger JF, McIver EE. 2003. Early Tertiary *Chamaecyparis* Spach from Axel Heiberg Island, Canadian High Arctic. *Canadian Journal of Botany-Revue Canadienne De Botanique* **81**: 113–130.

Kusumi J, Tsumura Y, Yoshimaru H, Tachida H. 2000. Phylogenetic relationships in Taxodiaceae and Cupressaceae sensu stricto based on *matK* gene, *chlL* gene, trnL-trnF IGS region, and trnL intron sequences. *American Journal of Botany* **87**: 1480–1488.

Lee C, Wen J. 2004. Phylogeny of *Panax* using chloroplast trnC–trnD intergenic region and the utility of trnC–trnD in interspecific studies of plants. *Molecular Phylogenetics and Evolution* **31**: 894–903.

Lewis PO. 2001. A likelihood approach to estimating phylogeny from discrete morphological character data. *Systematic Biology* **50**: 913–925.

Little DP, Schwarzbach AE, Adams RP, Hsieh C-F. 2004. The circumscription and phylogenetic relationships of *Callitropsis* and the newly described genus *Xanthocyparis* (Cupressaceae). *American Journal of Botany* **91**: 1872–1881.

Mao K, Milne RI, Zhang L, Penga Y, Liu J, Thomas P, Mill RR, Renner SS. 2012. The distribution of living Cupressaceae reflects the breakup of Pangea. *Proceedings of the National Academy of Sciences of the United States of America* **109**: 7793–7798.

Paull R, Hill RS. 2010. Early Oligocene *Callitris* and *Fitzroya* (Cupressaceae) from Tasmania. *American Journal of Botany* **97**: 809–820.

Peters MD. 1985. *A taxonomic analysis of a middle Cretaceous megafossil plant assemblage from Queensland, Australia.* PhD, University of Adelaide, Adelaide, SA, Australia.

Piggin J, Bruhl JJ. 2010. Phylogeny reconstruction of *Callitris* Vent. (Cupressaceae) and its allies leads to inclusion of *Actinostrobus* within *Callitris*. *Australian Systematic Botany* **23**: 69–93.

Pole M. 1998. Paleocene gymnosperms from Mount Somers, New Zealand. *Journal of the Royal Society of New Zealand* **28**: 375-–403.

Pole M. 2007. Early Eocene dispersed cuticles and mangrove to rainforest vegetation at Strahan-Regatta Point, Tasmania. *Palaeontologia Electronica* **10:** 15A:66p

Sakaguchi S, Bowman DMJS, Prior LD, Crisp MD, Linde CC, Tsumura Y, Isagi Y. 2013. Climate, not Aboriginal landscape burning, controlled the historical demography and distribution of fire-sensitive conifer populations across Australia. *Proceedings of the Royal Society B: Biological Sciences* **280**: 20132182.

Shaw J, Lickey EB, Beck JT, Farmer SB, Liu W, Miller J, Siripun KC, Winder CT, Schilling EE, Small RL. 2005. The tortoise and the hare II: relative utility of 21 noncoding chloroplast DNA sequences for phylogenetic analysis. *American Journal of Botany* **92**: 142–166.

Stockey RA, Kvacek J, Hill RS, Rothwell GW, Kvacek Z 2005. The fossil record of Cupressaceae *s. lat*. In: Farjon A ed. *A monograph of Cupressaceae and Sciadopitys*. Kew: Royal Botanic Gardens, 54–68.

Whang SS, Hill RS. 1999. Late Palaeocene Cupressaceae macrofossils at Lake Bungarby, New South Wales. *Australian Systematic Botany* **12**: 241–254.
